# Unraveling the Nature of Hydrogen Bonds of “Proton Sponges” Based on Car-Parrinello and Metadynamics Approaches

**DOI:** 10.3390/ijms24021542

**Published:** 2023-01-12

**Authors:** Beata Kizior, Mariusz Michalczyk, Jarosław J. Panek, Wiktor Zierkiewicz, Aneta Jezierska

**Affiliations:** 1 Faculty of Chemistry, Wrocław University of Science and Technology, ul. Wybrzeże Wyspiańskiego 27, 50-370 Wrocław, Poland; 2 Faculty of Chemistry, University of Wrocław, ul. F. Joliot-Curie 14, 50-383 Wrocław, Poland

**Keywords:** “proton sponge”, gas phase, crystalline phase, Hirshfeld surface, CPMD, PIMD, metadynamics, QTAIM, DORI

## Abstract

The nature of intra- and intermolecular non-covalent interactions was studied in four naphthalene derivatives commonly referred to as “proton sponges”. Special attention was paid to an intramolecular hydrogen bond present in the protonated form of the compounds. The unsubstituted “proton sponge” served as a reference structure to study the substituent influence on the hydrogen bond (HB) properties. We selected three compounds substituted by methoxy, amino, and nitro groups. The presence of the substituents either retained the parent symmetry or rendered the compounds asymmetric. In order to reveal the non-covalent interaction properties, the Hirshfeld surface (HS) was computed for the crystal structures of the studied compounds. Next, quantum-chemical simulations were performed in vacuo and in the crystalline phase. Car–Parrinello molecular dynamics (CPMD), Path Integral Molecular Dynamics (PIMD), and metadynamics were employed to investigate the time-evolution changes of metric parameters and free energy profile in both phases. Additionally, for selected snapshots obtained from the CPMD trajectories, non-covalent interactions and electronic structure were studied. Quantum theory of atoms in molecules (QTAIM) and the Density Overlap Regions Indicator (DORI) were applied for this purpose. It was found based on Hirshfeld surfaces that, besides intramolecular hydrogen bonds, other non-covalent interactions are present and have a strong impact on the crystal structure organization. The CPMD results obtained in both phases showed frequent proton transfer phenomena. The proton was strongly delocalized in the applied time-scale and temperature, especially in the PIMD framework. The use of metadynamics allowed for tracing the free energy profiles and confirming that the hydrogen bonds present in “proton sponges” are Low-Barrier Hydrogen Bonds (LBHBs). The electronic and topological analysis quantitatively described the temperature dependence and time-evolution changes of the electronic structure. The covalency of the hydrogen bonds was estimated based on QTAIM analysis. It was found that strong hydrogen bonds show greater covalency, which is additionally determined by the proton position in the hydrogen bridge.

## 1. Introduction

Molecular dynamics (MD) methods play a key role in studying changes in system properties as a function of time at the molecular level [1,2,3,4,5,6,7,8,9,10,11,12,13,14]. The classical MD simulations are realized by solving the corresponding Newton’s equations of motion for systems of interacting particles. The inter-particle forces and related potential energies are often calculated based on interatomic potentials or classical molecular mechanics force fields. These methods are used in chemical physics, biophysics, chemistry and materials science [15,16,17]. One of the most important methods belonging to ab initio time-evolution schemes is Car–Parrinello Molecular Dynamics (CPMD) [2]. The main idea of the CPMD method is to separate the fast motion of electrons from the slow motion of atomic nuclei on a time scale, and then replace the dynamics of real electrons with the dynamics of fictitious particles—Kohn-Sham orbital coefficients.The slow motion of atomic nuclei is then characterized based on the classical (Newtonian) mechanics. In turn, the fast motion of electrons (or rather changes in the orbital composition) is determined by the Density Functional Theory (DFT) with reference to the Kohn–Sham equations [18]. This approach allows a detailed discussion of effects related to temperature or pressure as well as entropic changes in the investigated chemical species. This method allows a satisfactory description to be obtained of the nature of interactions in the studied systems. Thus, it is one of the methods applied very often to obtain a detailed characteristic of intra- and intermolecular interactions; see, e.g., Refs. [19,20,21,22,23,24,25,26,27,28,29,30,31]. These interactions play a fundamental role in chemistry, biochemistry, and biology in determining the structure and physico-chemical properties of systems [32,33,34]. In order to describe the quantum nature of nuclei, Path Integral Molecular Dynamics (PIMD) was developed [6,35]. The method was successfully applied in our numerous studies of intra- and intermolecular interactions [23,36,37].

The presence of intramolecular hydrogen bond results very often in the quasi-ring formation, which stabilizes the spatial structure of compounds [31,38]. Other examples could be the catalysis of reactions involving enzymes and proteins [39]; the formation of secondary, tertiary, and quaternary structures in proteins [40,41]; and the modulation of the electronic structure and aromaticity of compounds [42,43]. It is well known that hydrogen bonds play an important role in proton transfer (PT) processes [44,45], as well as in interactions during charge transfer [46]. Moreover, they can change and affect the resonance capabilities of the substituents (electron-donating or -accepting) in the molecules [42]. Hence, it can be said that the role of intramolecular hydrogen bonding is very significant.

**Figure 1:**
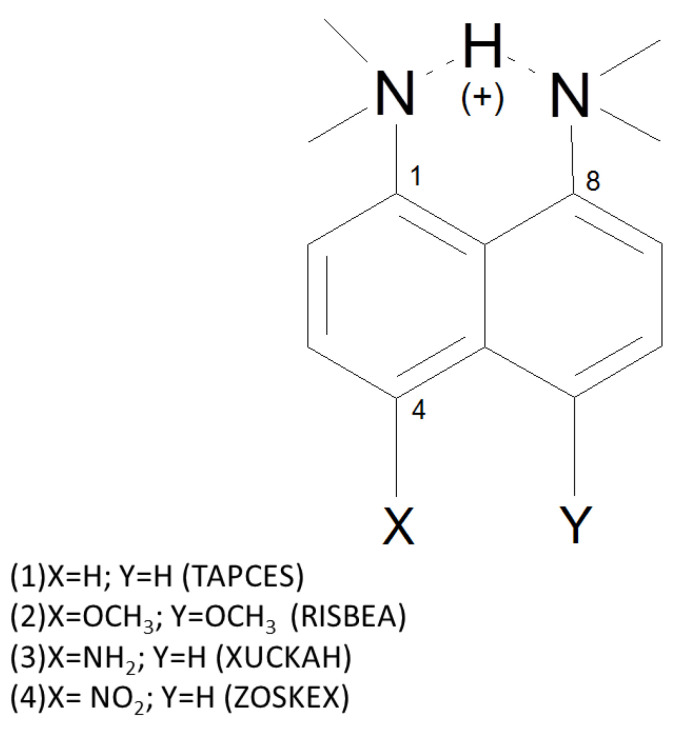
Structure with CCDC code and relevant atoms numbering of the protonated forms of (**1**) 1,8-bis(dimethylamino)naphthalene (TAPCES); (**2**) 1,8-bis(dimethylamino)-4,5-dimethoxynaphthalene (RISBEA); (**3**) 4-amino-1,8-bis(dimethylamino)naphthalene (XUCKAH); (**4**) 4-nitro-1,8-bis(dimethylamino)naphthalene (ZOSKEX). The dotted lines indicate the presence of intramolecular hydrogen bonds.

In the current study, quantum-chemical results for the protonated form of 1,8-bis(dimethyl- amino)naphthalene (DMANH${}^{+}$) and its three derivatives (see Figure 1 and Figure S1 of the Supplementary Materials) are reported [47,48,49,50]. These compounds are known as “proton sponges” in the literature. The first report characterizing 1,8-bis(dimethylamino)naphthalene (DMAN) was published in 1968 by Alder et al. [51]. The DMAN molecule contains two NMe${}_{2}$ groups in close proximity, so the attachment of the proton leads to the formation of a short intramolecular N-H⋯N hydrogen bond. Recent studies indicate that N⋯N distance can change in the range of 2.522–2.621 Å [52,53,54,55,56,57,58]. It should be noted that the shortest N⋯N distances are found in systems with a symmetric HB [53,54,55,57,59,60]. The intramolecular HB in the protonated form of 1,8-bis(dimethylamino)naphthalene has been described as a strong Low-Barrier Hydrogen Bond (LBHB) [27,61,62]. In the literature it was reported that LBHB can be of a single or double potential minimum type [63]. The IR spectra recorded for “proton sponges” exhibit an extremely low frequency absorption band in the 500–600 cm${}^{-1}$ region, indicating the presence of N-H⋯N moiety [64]. In turn, the NMR spectroscopy ${}^{15}$N–${}^{15}$ N chemical coupling constant (${}^{2}$J${}_{NN}$) of the intramolecular symmetric HB of the “proton sponge” has a value of 11 Hz [64,65,66]. In addition to their spectroscopic features, these systems exhibit excellent base properties [67,68,69]. The first reported value of pKa for 1,8-bis(dimethylamino)naphthalene (DMAN) was 12.34 [51]. Various proton sponges have been synthesized with much better base properties than DMAN until now [62,68,70]. Therefore, it can be concluded that proton sponges are an interesting group of compounds to study. These features often receive credit in materials chemistry [71], as well as in catalysis, such as in the activation of CO${}_{2}$ [72]. A relatively small number of available experimental and theoretical studies, as well as short interatomic separation in the N-H⋯N moiety [64], large values of the ${}^{15}$N–${}^{15}$N coupling constants [64,65,66], and other interesting physico-chemical features of DMANH${}^{+}$ and its derivatives, prompted us to investigate the dynamic nature of the intramolecular hydrogen present in the investigated “proton sponges” (see Figure 1).

These types of studies are a valuable source of information used in crystal engineering for the rational design of new systems with the desired physico-chemical properties and structural features. Following this line of thinking, our work focused on the theoretical investigation of intramolecular (N-H⋯N) hydrogen bonds in the gas and crystalline phases. The Hirshfeld surface (HS) [73,74] and fingerprint [75,76] approaches were used to reflect intra- and intermolecular interactions in the crystal structures. Quantum-chemical simulations were carried out using Car–Parrinello Molecular Dynamics (CPMD) [2]. Finally, the Quantum Theory of Atoms in Molecules (QTAIM) and Density Overlap Regions Indicator (DORI) approaches were used to obtain detailed characteristics of intramolecular hydrogen bonding and non-covalent interactions in the prototypic symmetric DMAN compound, its methoxy derivative, and asymmetric derivatives—substituted by amino and nitro group, respectively. This study aims to deepen the understanding of the nature of intramolecular interactions in the particular “proton sponges” and to answer the following questions:

(i) What is the mechanism of proton motion in an intramolecular N-H⋯N bond in symmetric/asymmetric “proton sponges”?
(ii) Does the proton transfer play a determinant role in changing the physico-chemical features in selected “proton sponges”?
(iii) What is the environmental impact on the intramolecular hydrogen bond features?
(iv) What is the temperature impact on the dynamics of the intramolecular hydrogen bond?

To the best of our knowledge, this is one of the few attempts to use Car–Parrinello Molecular Dynamics (CPMD) [2] and Path Integrals Molecular Dynamics (PIMD) [6,35] to study HB dynamics in symmetric and asymmetric “proton sponges”. One of our earlier studies [29] was devoted to the CPMD investigation of proton dynamics in symmetric “proton sponges” including DMANH${}^{+}$, but in the gas phase only and with emphasis on the vibrational signatures. The particular feature of the current work is a comparison of dynamical nature of the intramolecular hydrogen bond and its properties as a function of time in two different environments—gas phase vs. crystalline phase—and of temperatures.

## 2. Results and Discussion

### 2.1. Hirshfeld Surface Analysis

**Figure 2:**
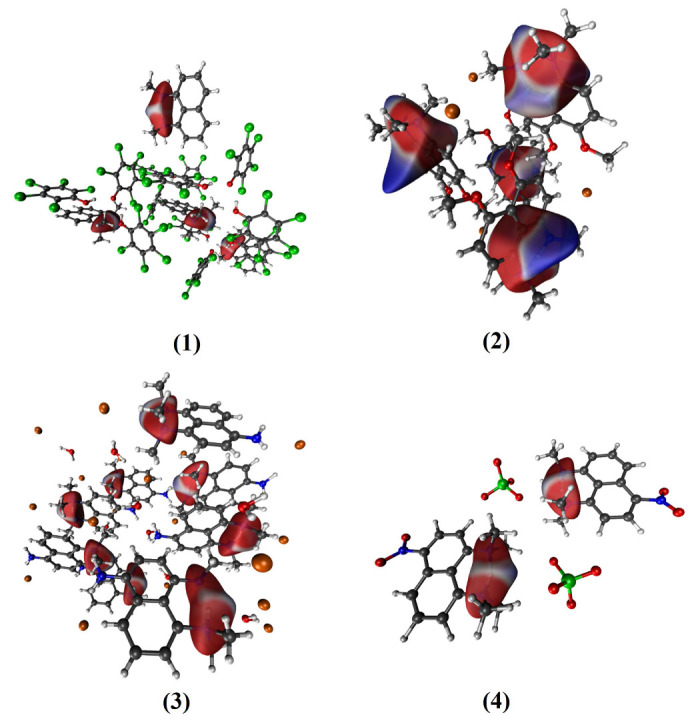
Hirshfeld surfaces obtained for the studied “proton sponge” crystals with emphasis on intramolecular hydrogen bonding. Color coding: red—oxygen; blue–nitrogen; gray–carbon; white—hydrogen; green—chlorine; orange—bromine. (**1**)–(**4**) denote the studied compounds.

**Figure 3:**
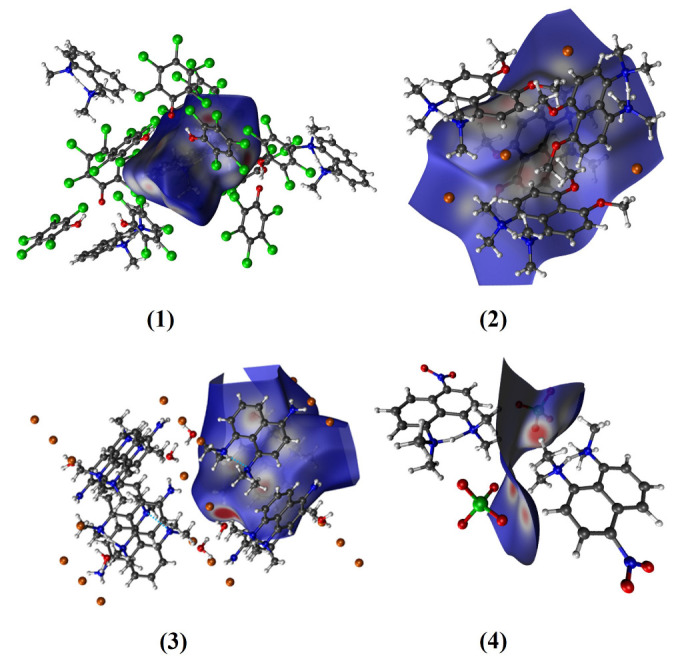
Hirshfeld surfaces obtained for the studied “proton sponge” crystals with emphasis on intermolecular interactions. Color coding: red—oxygen; blue—nitrogen; gray—carbon; white—hydrogen; green—chlorine; orange—bromine. (**1**)–(**4**) indicate the studied compounds.

The Hirshfeld surface (HS) analysis of the investigated symmetric and asymmetric “proton sponges” was carried out and are illustrated in Figure 2 and Figure 3 (see Figure S2 for relevant unit cells). The size and shape of the Hirshfeld surface allow us to study and show qualitatively intra- and intermolecular interactions in molecular crystals [73,77]. This analysis describes the d${}_{e}$ (distance from the Hirshfeld surface to the nearest atom outside the surface), d${}_{i}$ (distance from the Hirshfeld surface to the nearest atom inside the surface) as well as d${}_{norm}$ (normalized contact distance) in crystal structures [73,74]. In Hirshfeld maps, the interactions with a distance equal to the sum of the van der Waals (VDW) radii are illustrated with the white surface. In turn, red areas characterize shorter interactions, and blue indicates interactions with distances longer than the VDW radii. The red surface around the two nitrogen atoms represents the negative potential (the acceptor), and the blue area around the hydrogen atoms illustrates the positive potential (the donor). The formation of a quasi-ring due to the presence of intramolecular HB has a significant impact on the structure and molecular properties of the studied compounds. It is worth noting that blue areas were revealed for compound (**2**), indicating the presence of interactions with distances greater than VDW radii. From the HS analysis results, we can conclude that the intermolecular interactions in the crystal structures play a key role in the crystal stability, as presented in Figure 3 and Figure S3 in the Supplementary Information.

In the analyzed symmetric and asymmetric “proton sponges”, the molecules are organized in the crystal through Cl⋯H and O⋯H, H⋯C and O⋯H, O⋯H, H⋯C and H⋯Br, O⋯H short contacts for compounds (**1**)–(**4**), respectively, (see Figure 3 and Table 1).

It was found that the shortest O⋯H contacts were present in compounds (**3**) and (**4**), as indicated by the red areas (see Figure 3). The different trend was noted for symmetrical compounds, where the areas characterizing the O⋯H interaction are much lighter. Thus, the molecular packing of the studied crystals is strongly influenced by the presence of intra- and intermolecular interactions. In the next step, the fingerprint plot approach was used to investigate short and long contacts in the studied crystals, as presented in Figure S3 in the Supplementary Information. The fingerprint maps show a picture similar to HS. The plot in Figure S3 shows the usual features confirming the presence of an intramolecular hydrogen bond. The obtained plots for the “proton sponges” contain expected intramolecular HB spikes at the bottom left of the maps (the lower spike, d${}_{i}$ > d${}_{e}$) (see Figure S3). All crystal structures exhibit diverse O⋯H contacts. Interestingly, the crystal structures of compounds (2) and (3) show “wings” in the upper left corner which proves the presence of C-H⋯π interactions in the studied structures and is consistent with the results obtained for the naphthalene ring [75]. Compounds (1) and (4), on the other hand, exhibit this property to a far lesser extent. In summary, the presented results allowed us to conclude that intramolecular hydrogen bonding is present in all symmetric/asymmetric “proton sponges” taken into consideration in the study. In addition, intermolecular interactions are fundamental for maintaining stability in the crystal structures of the compounds.

### 2.2. Car–Parrinello Molecular Dynamics (CPMD) in the Gas and Crystalline Phases

The electronic structure framework behind the Car–Parrinello Molecular Dynamics (CPMD) scheme, namely Density Functional Theory (DFT), has profound advantage over the classical force fields: it allows for a description of the bond breaking and forming processes without any special provisions. Thus, the DFT-based CPMD is ideally suited for the studies of proton dynamics in hydrogen bonds, where short-lived or permanent proton transfer (PT) events can occur, and where a priori knowledge of proton positions is not always possible.

**Figure 4:**
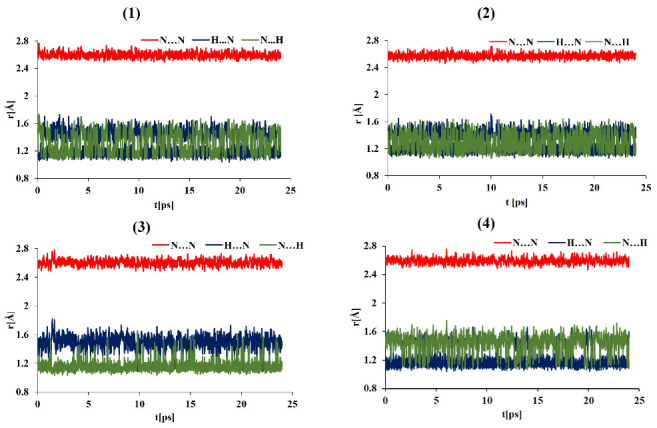
Time evolution of the intramolecular hydrogen bond metric parameters. Gas phase CPMD results at 100 K for DMANH${}^{+}$ and its derivatives.

**Figure 5:**
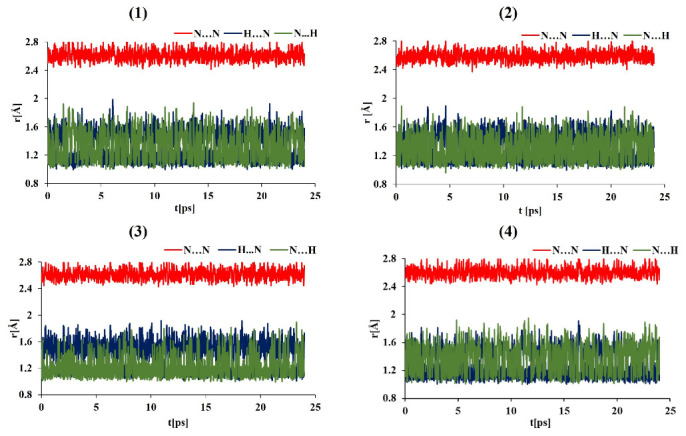
Time evolution of the intramolecular hydrogen bond metric parameters. Gas phase CPMD results at 300 K for DMANH${}^{+}$ and its derivatives.

**Figure 6:**
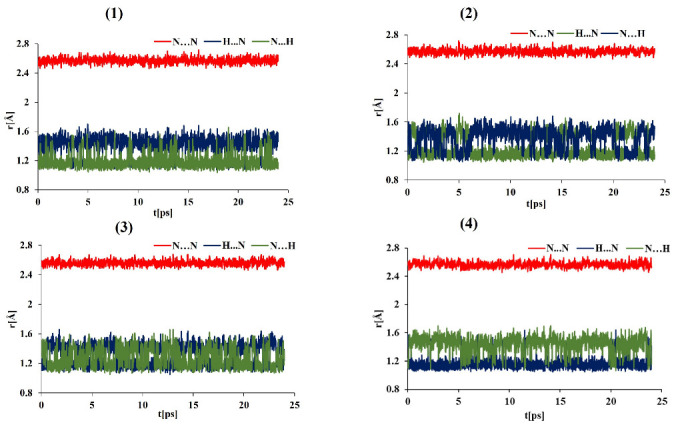
Time evolution of the intramolecular hydrogen bond metric parameters. Crystalline phase CPMD results at 100 K for DMANH${}^{+}$ and its derivatives.

**Figure 7:**
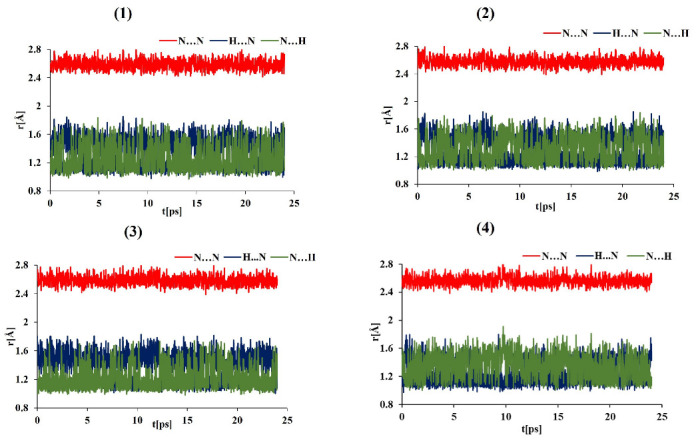
Time evolution of the hydrogen bond metric parameters. Crystalline-phase CPMD results at 300 K for DMANH${}^{+}$ and its derivatives.

The data on the evolution of the relevant N-H⋯N bond lengths as functions of time are shown in Figure 4, Figure 5, Figure 6, Figure 7 and Figures S4–S7 (for the OLYP functional) in the Supplementary Information. In order to obtain detailed differences in the dynamics of the bridge protons for asymmetric and symmetric “proton sponges”, the CPMD simulations were performed in the gas and crystal phases at 100 K and 300 K using two functionals (PBE and OLYP). Figure 4 and Figure 5 show that for compounds (1)–(3), the bridge proton is located on the donor (N) side in the gas phase simulations. Interestingly, in the investigated compounds (1)–(3), the bridged proton does exhibit very often short contacts with the acceptor nitrogen (N) atom; therefore, it can be indeed described as very labile. The situation is different for an asymmetric “proton sponge” with the nitro group as the substituent (compound (4)), in which the proton present in the intramolecular hydrogen bridge is more often observed on the acceptor side (see Figure 4 and Figure 5). At this point, it is worthwhile to notice that the notions of the “donor” and “acceptor” are not well defined, because the neutral “proton sponge” catches the proton between the two nitrogen atoms, so we have chosen to denote as “donor” the nitrogen atom at position 1 of the naphthalene moiety. In the case of the asymmetric proton sponges, one could use the diffraction data to assign the “donor” and “acceptor” labels, but we prefer to use a consistent notation throughout this work. One last observation from the gas phase results: the events of proton entering the acceptor site are more frequent for the symmetric “proton sponges” (1) and (2).

The crystal environment seems to promote instantaneous proton sharing or proton transfer events, which are visibly more frequent in Figure 6 and Figure 7 than in Figure 4 and Figure 5. Compound (4) consistently exhibits dominant proton position differently from the compounds (1)–(3). However, the semi-quantitative nature of the distance timeline graphs prevents further detailed analysis solely on their basis. Before proceeding with statistical analysis of the trajectories, it is interesting to observe that the time evolution data for the hydrogen bridge structural parameters are very similar for the two proposed functionals in both environments and temperatures. Thus, the applied computational level satisfactorily reproduced the evolution of the proton in the hydrogen bridge as a function of time. In summary, the influence of environment and temperature plays a key role in our CPMD study in the gas and crystal phases, as they were able to alter the dynamics of bridged protons, introducing events not observed for the isolated molecule. A quantitative description of these phenomena was carried out on the basis of proton possession statistics.

Proton possession, defined as the percentage of the simulation time spent by the proton within either the donor or the acceptor site region, is an important characteristics of the symmetry of the proton potential energy well. In the current study, the simplest and most intuitive definition is used: the proton is in (temporary) possession by that of the two nitrogen atoms to which it is closest. As already noted above, the donor and acceptor concepts are not well established for the strong hydrogen bonding in the “proton sponges”; therefore, the data in Table 2 and Table S1 was calculated with reference to the nitrogen atom at the position 1 of the aromatic moiety. At the beginning of this part of data analysis, it should be emphasized that the results for the PBE functional reported in the article (Table 2) are in very close agreement with the OLYP functional calculations (reported in the Supporting Information, Table S1).

Comparison of the statistics in Table 2 for different temperatures revealed that the barrier between two potential energy minima is sufficient to severely hinder the proton mobility at 100 K. There are two possible sources of the perceived asymmetry of the proton possession data: either the minima are equivalent but the barrier cannot be easily crossed at 100 K (so that the proton is trapped in one of the minima), or the minima are in fact not equivalent. The former option happens in the gas phase, while the latter can result from the asymmetry of the crystal environment. The gas phase results for the symmetric compounds (1) and (2) reflect the effect of the barrier height by deviating from the ideal 50–50% proton possession. This deviation is larger at 100 K, up to 6% and up to 2.1% at 300 K. The influence of the crystalline environment is very significant at 100 K—the compounds which are symmetric by themselves suddenly lock the proton in one of the potential wells for ca. two thirds of the simulation time. This effect is much smaller at 300 K, where again the proton potential profile is approaching the symmetric double well shape. The situation is very different when the asymmetric compounds (**3**) and (**4**) are analyzed. The gas phase results are indicative of the substituent effect only, and the impact of two selected substituents at position 4, namely the -NH${}_{2}$ group in (3) and the -NO${}_{2}$ group in (4), is clearly significant indeed. While substitution by another amino group keeps the proton at the same ring where the additional -NH${}_{2}$ functional group is present, the -NO${}_{2}$ substitution strongly promotes the proton transition to the other half of the naphthalene moiety. An increase in the temperature from 100 K to 300 K makes the proton distribution more balanced by ca. 20%, e.g., from 91.5% to 74.2% for (3). The asymmetry of the preferred proton location agrees well with the substituent properties. The electron-withdrawing nitro group in position 4 makes position 1 more positively charged and thus less preferable to the proton. On the other hand, the electron-donating -NH${}_{2}$ group at position 4 increases electron density at position 1, making the proton more willing to reside at this site. Interestingly, the crystalline phase results for (3) and (4) at 300 K are so similar to their gas phase counterparts that the effect of neighboring moieties seems to be non-existent. However, upon lowering the simulation temperature to 100 K, we found out that this effect is very significant for (3), counteracting the intramolecular modulation of the proton position. These findings underline the delicate nature of the interplay between intra- and inter-molecular factors determining the proton potential function in the studied compounds.

### 2.3. Path Integral Molecular Dynamics—Inclusion of Nuclear Quantum Effects

**Figure 8:**
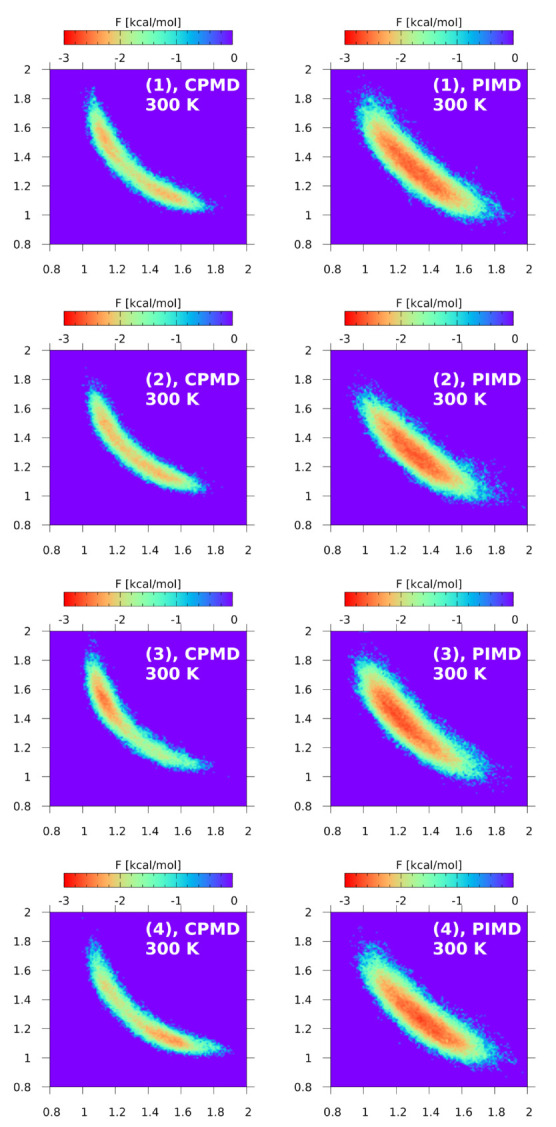
Free energy surface for proton motion in the compounds (1)–(4)-comparison of gas phase simulations at 300 K with classical (CPMD) and quantum (PIMD) nuclear regime. The axes are the N⋯H distances—the horizontal axis for the donor-H and the vertical axis for the acceptor-H distances in Å.

Car–Parrinello Molecular Dynamics is inherently classical (Newtonian) when the nuclear degrees of freedom are considered. However, light nuclei such as protons can exhibit strong nuclear quantum effects, including tunneling and probability distribution broadening. These effects can be efficiently sampled within the Path Integral Molecular Dynamics (PIMD) [6] framework. A serious drawback of PIMD is that it does not provide access to real-time quantum dynamics of the system; the ensemble averages are the properties that reflect the inclusion of quantum effects. Therefore, the results of PIMD cannot be presented as time evolution data such as in Figure 4, Figure 5, Figure 6 and Figure 7. We have converted both CPMD and PIMD trajectory data from the gas phase simulations at 300 K into probability distributions ρ(r), and then—via the formula F(r) = –$k_{B}T$lnρ(r)—we have arrived at the free energy landscapes presented in Figure 8. The landscapes are prepared as functions of the N-H distances in the intramolecular hydrogen bonds of the studied systems. The horizontal axis in each subfigure of Figure 8 corresponds to the “donor”-proton N–H distance, while the vertical one corresponds to the proton–“acceptor” distance.

Figure 8 shows that the asymmetry of the proton positions is visible for the molecules of (3) and (4) with classical (CPMD) description of nuclei. There is also a small difference between the CPMD results for (1) and (2)—the prototypic DMANH${}^{+}$ (1) seems to have a small barrier for the proton transfer. When the nuclear quantum effects are included via PIMD, a significant broadening of the proton distribution is observed, and careful insight allows us to state that the N⋯N distance was shortened by quantum effects because the outer regions of the probability distributions for PIMD end at lower N—H distances than for CPMD. The quantized protons are located closer to the center of the bridge than in the CPMD, especially for the compounds (1)–(2). In these cases, the free energy lansdcape seems to be barrierless and effectively corresponds to a single-well profile. The asymmetry of the proton distribution is still visible for (3)–(4) and is in agreement with the proton possession data presented in the previous section.

The reported free-energy landscapes exhibit small coverage of the phase space, but, given the sterical restraints of the tight hydrogen bond of proton sponges, this is not surprising. The well depths were estimated at ca. 3 kcal/mol, which definitely does not correspond to the energy of strong, sterically and electronically assisted hydrogen bonds. This is, however, the result of relying on thermal energy only. Reproducing the hydrogen bond energy in detail requires the use of “accelerated sampling” techniques. One of such schemes is metadynamics, and its application to the proton sponges is presented below.

### 2.4. Metadynamics

The CPMD and PIMD results presented in the previous sections showed a significant range of modulation of the proton position in the hydrogen bond depending on substitution, the electrostatic field of the crystal, and the temperature. The metadynamics [78] study is aimed at reproducing the free energy surface for proton motion with a history-dependent potential so that the barriers can be easily crossed and their heights estimated more accurately than for standard CPMD. The latter case can utilize only the thermal energy to cross the barriers, while metadynamics is an extended theory aimed at overcoming the barriers via added Gaussian hills gradually filling up the current potential well.

**Figure 9:**
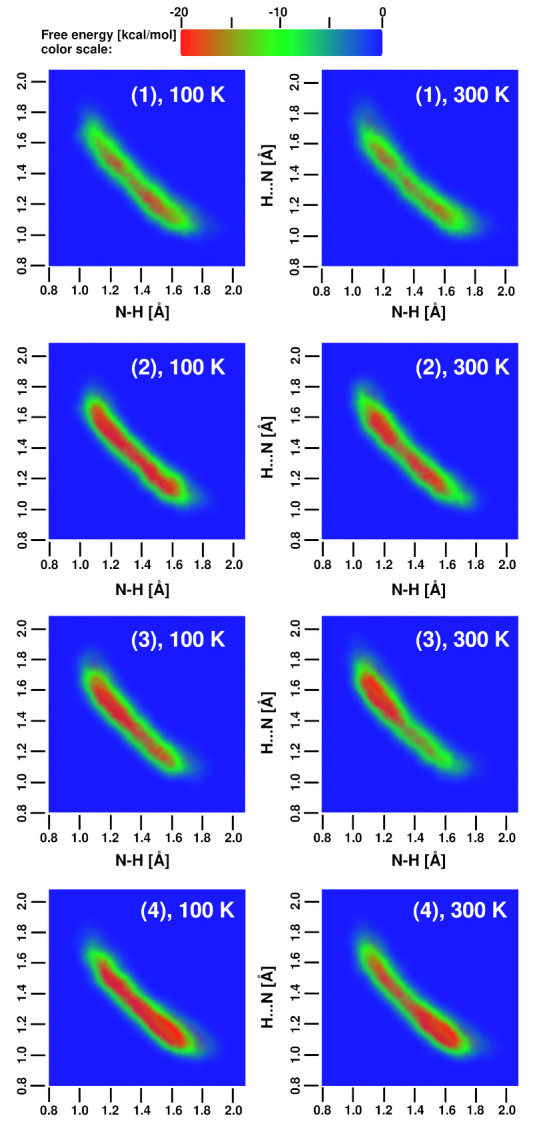
Free energy surface for proton motion in the compounds (1)–(4). Two-dimensional slices across the three-dimensional volumetric data resulting from the CPMD-based gas phase metadynamics. The N⋯N value for the slice is 2.6 Å.

The results for the gas phase metadynamics for compounds (1)–(4) are shown in Figure 9. The metadynamics was carried out in the phase space of three variables, e.g., N-H, H⋯N, and N⋯N distances, while the figure shows slices across the N⋯N axis. The chosen N⋯N value of 2.6 Å corresponds well to the average N-H distance in the studied compounds. The depths of the well vary significantly, from only −17.7 kcal/mol for (1) to −34 kcal/mol for (4). However, we have chosen in the Figure to describe the range of free energy from −20 to 0 kcal/mol, that is the fragment of the potential hypersurfaces corresponding to the most abundant N⋯N region. This has some bearing on the shape of the surfaces, especially for (4) where the true depth is larger, but this convention allows for easy comparison of the energy profiles. Starting from the prototypic DMANH${}^{+}$, compound (1), one can note that the free energy wells are different between 100 K and 300 K. The minima at 100 K are deeper, which is not surprising—three times smaller kinetic energy values than for 300 K allow for much less frequent visits of the proton to the acceptor side. Crossing the minima (lying at −17.7 kcal/mol) requires increase in the free energy to −10 kcal/mol, which turns out not to be too problematic even at 100 K, as seen in Figure 4 and in the proton possession data described above. In case of the compound (2), the minima are deeper, −22 kcal/mol, but still symmetrical. Raising the well bottoms at 300 K reveals the barrier at the middle of the bridge, i.e., at equal N-H and H⋯N distances (1.4 Å). The most curious are the cases of (3) and (4). Compound (3) has a strongly asymmetric well, biased towards the “donor” atom. On the contrary, the molecule of (4) tends to place the proton at the “acceptor” side, e.g., the ring devoid of substituents, with the depth reaching −34 kcal/mol. The reported depths of the free energy surface for all four compounds, from −17.7 to −34 kcal/mol, show indeed that the proton is strongly held by the NMe${}_{2}$ groups forming characteristic “clamps”, and the shallow barriers indicate that the involved hydrogen bonds are of the LBHB class. It should be mentioned at the end of this section that even after introducing a significant amount of energy into the system, the metadynamics did not lead to the event of the proton being pushed out of the hydrogen bond area. The potential energy pumped into the system via the metadynamics quite often flows into unexpected channels, and the fact that the proton stays in the N-H⋯N bridge is one more manifestation of the specific mechanism of basicity of the “proton sponges”.

### 2.5. Static Density Functional Theory (DFT)

For the discussed “proton sponges”, the reaction path describing the proton transfer in the hydrogen bond was investigated. The simulations were performed at the PBE0-D3/def2-TZVP level of theory. The obtained results are shown in Figure S8. The relative energy barrier is less than 1 kcal/mol in all studied cases. Two energy minima were detected for each of the studied compounds. In the asymmetric molecules, the secondary minimum (located at the “acceptor” site for (3) and the “donor” site for (4), respectively) is ca. 0.5 kcal/mol above the primary minimum. Such a low energy barrier indicates that the bridged proton can easily move between the donor and acceptor atoms. The static DFT results correspond well with the CPMD, PIMD, and metadynamics findings.

**Figure 10:**
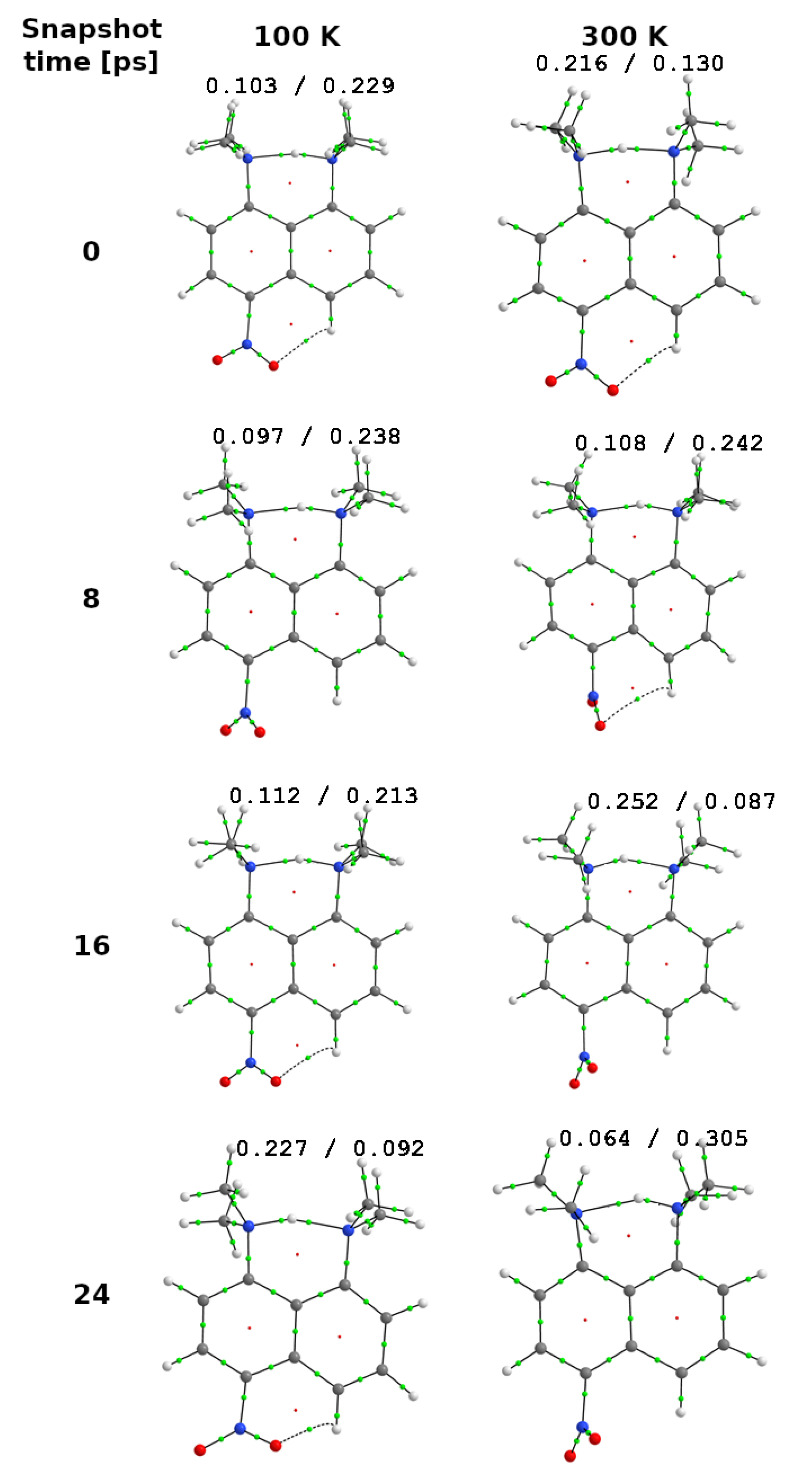
QTAIM molecular diagrams for compound (4) at 100 K and 300 K. Green dots represent bond critical points (BCPs) while red – ring critical points (RCPs). Values of electron densities at the left and right N-H BCPs are given in a.u. Color coding: red—oxygen; blue—nitrogen; gray—carbon; white—hydrogen.

The QTAIM parameters for the studied molecules are gathered in Table S2. Two bond critical points (BCPs) for N⋯H and H⋯N interactions, including protons fluctuating between dimethylamino (-NMe${}_{2}$) groups, were considered for these systems. Differences between these parameters demonstrate which NMe${}_{2}$ moiety the proton belongs to at a given time. At first glance, one may notice that all values of ρ at BCPs are in approximate range of 0.1–0.3 a.u., which signifies the certain amount of covalency in all the investigated interactions. The behavior of protons, which balances between two nitrogen atoms, is expressed by the difference in values presented in single-table cells for a given compound at 100 K or 300 K temperature. It must be recalled that in compounds (1) and (2), the dimethylamino groups attached to the ring are indistinguishable due to the high symmetry of the molecules. In turn, in compounds (3) and (4), the data in the upper row of a single table cell refer to the N⋯H interaction with the N atom of the -NMe${}_{2}$ group attached to the substituted ring (in the sections above denoted as the “donor”), while the data in lower row concerns H⋯N interaction where N atom is derived from the moiety incorporated into the unsubstituted ring (denoted as the “acceptor”). For clarity, the QTAIM molecular diagram for compound (4) in four snapshot time projections is captured in Figure 10. For this compound at 100 K, one can see that in the first three snapshots (0, 8, and 16 ps), the proton is shifted into the acceptor nitrogen closer to the ring without the nitro functional group. It is represented by the values of ρ of about 0.2 a.u. in a lower row towards the ones of about 0.1 a.u. appearing in upper row. This is followed by other QTAIM parameters: for the stronger H⋯N interactions, the value of $\nabla^{2}\rho$ is negative (as opposed to a positive or minimally negative value of this parameter for a weaker N⋯H interaction), the ϵ (ellipticity) is lower (from 0.006 to 0.009), which means a lower amount of π component in this bond, and its higher dynamic stability, H (electron energy density), is more negative and finally the |V|/G ratio (potential energy density and kinetic energy density, respectively) is greater (significantly exceeds 1 while for the weaker contact, it is only slightly higher than 1). The combination of H, V, and G parameters is in line with the remaining QTAIM descriptors and accords with Bader’s QTAIM protocol indicating that the stronger H⋯N interaction is covalent in nature, while the weaker one admittedly bears some hallmarks of covalency but is much weaker. The situation is completely reversed for the fourth snapshot, where the QTAIM parameters point to the supremacy of the H⋯N interactions with hydrogen directed to the nitrogen inserted in amino group of the substituted ring. When switching temperature to 300 K, the proton transfer between -NMe${}_{2}$ groups can be easily identified. At 0 and 16 ps, the one type of hydrogen bond is favored, while at 8 and 24 ps, the second one dominates. Similar patterns can be deduced for remaining compounds in different temperatures. As a general conclusion regarding QTAIM studies at 300 K, the proton motion is faster in this temperature as changes in the affiliation of hydrogen to interact with a particular nitrogen atom become more rapid than at 100 K. Only in a few cases can one consider the proton in a state of “suspension” between both competing groups. The compound (3) at 100 K captured at 8 ps can serve as such an example. All relevant quantities are identical or very similar for both BCPs in this situation.

These results can be put into the context of earlier studies of intermolecular [NHN]${}^{+}$ bridges. Literature reports classified such structural motif as covalent bond between the one of the N atoms and hydrogen atom (N-H) along with the second, significantly weaker contact, which was treated as a relevant hydrogen bond (H⋯N). Recently, in the work of El-Emam et al. [79] the N-H⋯N hydrogen bond was found in the crystal structure of adamantane-1,3,4-thiadiazole hybrid derivatives. Despite being formally classified as a non-covalent interaction, the ρ at BCP connecting the analyzed atoms was in the range of 0.173–0.220 a.u. (near to our findings for the majority of the current cases). Therefore, it exhibits distinguishing characteristics of a shared bond. Other factors, including the positive Laplacian of electron density, H(r) < 0, and -V/G ratio > 1, show that the intermolecular N-H-N contact in this system has a bonding type that is halfway between shared and closed-shell interaction. Two additional investigations that are comparable to ours in terms of the type of N-H contact examined solely theoretical models. Deepa et al. [80] found that the N-H⋯N hydrogen bonds between the AT and GC nucleic acid base pairs and selected amino acid side chains were relatively weak. The ρ was between 0.032 and 0.049 a.u., and other parameters also took on values typical of weaker non-covalent interactions, namely $\nabla^{2}\rho$ >0 and an ε oscillating around 0.050–0.060, which are very far from the results obtained in the current work. Finally, Bavafa et al. [81] studied the interactions between nitrosamine and formamide or formic acid. The QTAIM analysis unveiled the presence of an NHN bridge in which the proton was probably shifted into one of the nitrogen atoms. The partition of this fragment into a strong covalent bond and a weak non-covalent contact was clearly indicated by the ratio of the ρ values at the proper BCPs, which was 0.317 to 0.0213 a.u. The Laplacian of ρ had a negative sign for a stronger one, while a positive sign for a weaker one. The H value had the reverse relationship; it was positive for the unshared interaction.

**Figure 11:**
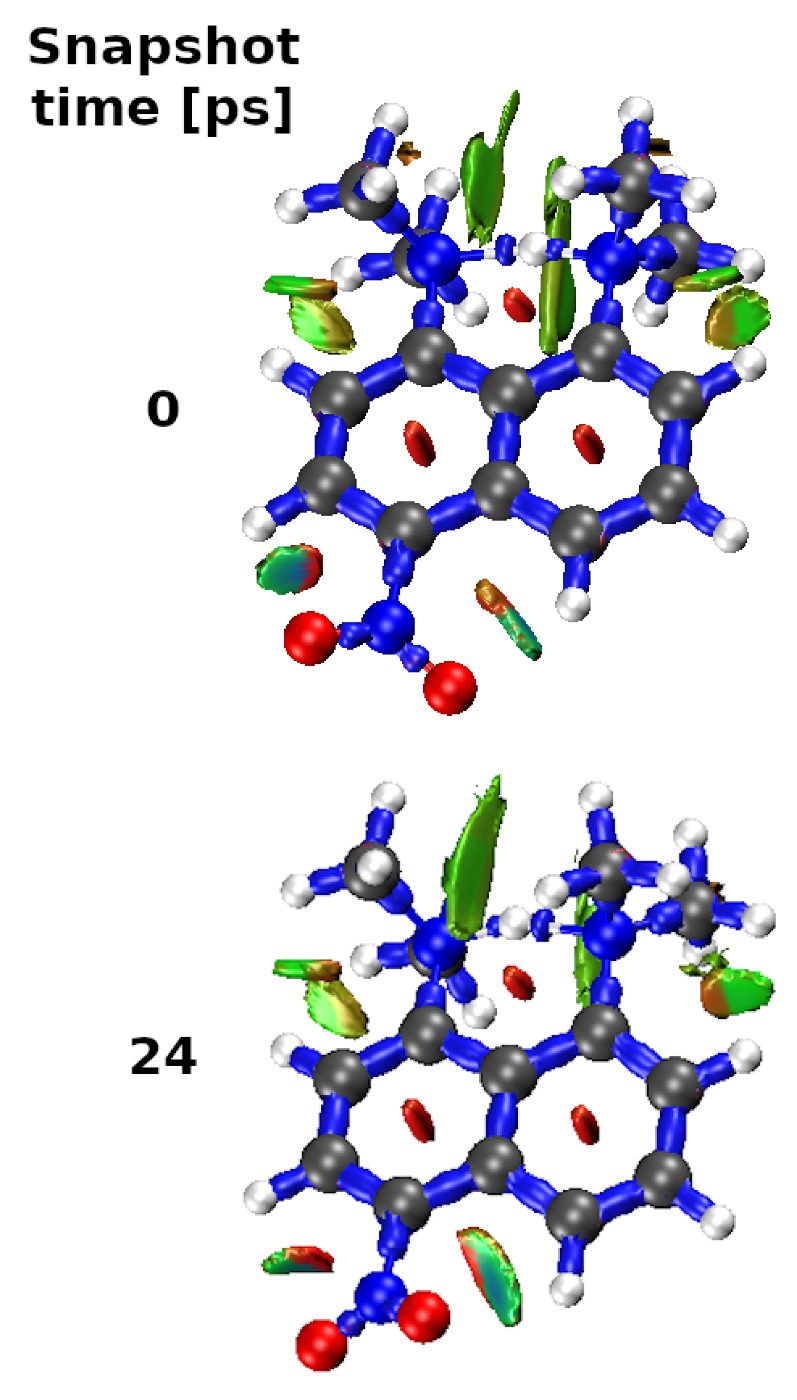
DORI isosurfaces (green spheres represent non-covalent interaction regions, blue s spheres represent covalent ones, and red spheres represent steric repulsions) in the RDG 0.5 a.u. isovalue for compound (4) at 100 K at 0 and 24 ps.

Unfortunately, the non-covalent index (NCI) analysis was unable to identify the studied interactions because of their high strength. Therefore, alternatively, the DORI approach [82] was applied for compound (4). The DORI molecular diagrams in Figure 11 illustrate the covalent (blue) and non-covalent (green) interacting regions for compound (4) at 100 K temperature at 0 and 24 ps. As can be seen from this figure, the whole N-H-N bridge is treated as stabilized by covalent interactions of comparable magnitude as those between carbon atoms in the ring or N-O bonds in the nitro group. The green regions signifying non-covalent contacts are shown in the case of weak O⋯H hydrogen interactions.

## 3. Materials and Methods

Hirshfeld surface (HS) [73,74] and fingerprint plot [75,76] methods were used to visualize and verify intra- and intermolecular interactions in the crystal structures of the investigated compounds—symmetric/asymmetric “proton sponges” [47,48,49,50]. These analyses were performed in the MultiWFN program [83,84]. Car–Parrinello molecular dynamics [2] was carried out using the CPMD 4.3 program [85]. Simulations were performed in the gas and crystalline phases. The models for the CPMD crystalline phase studies were prepared on the basis of crystal structures deposited in the Cambridge Crystallographic Data Centre (CCDC) [86] denoted as **TAPCES** with the deposition number 1266338 (1-dimethylammonio-8-dimethylaminonaphthlene pentachlorophenolate bis(pentachlorophenol) [47], **RISBEA** with the deposition number 122006 (1,8-bis(dimethylamino)-4,5-dimethoxynaphthalene hydrobromide) [48], **XUCKAH** with the deposition number 170012 (4-amino-1,8-bis(dimethylamino)naphthalene hydrobromide monohydrate) [49], and **ZOSKEX** with the deposition number 1315231 (4-nitro-1-dimethylamino-8-dimethylammonionaphthalene perchlorate) [50] (see Figure S2 in Supplementary Information and Table 3 for details). The gas-phase models (single molecules) were prepared using X-ray coordinates as well and were placed in cubic boxes with a = 12 Å (TAPCES), a = 15 Å (RISBEA), a = 14 Å (XUCKAH), and a = 14 Å (ZOSKEX), respectively. The DMANH${}^{+}$ derivatives include either electron-donating (-NH${}_{2}$ and -OCH${}_{3}$) or -accepting (-NO${}_{2}$) substituents attached in the para position to the naphthalene ring relative to the NMe${}_{2}$ group (see Figure 1).

In the first step, all prepared models were optimized with the Hessian matrix initialized according to the Schlegel scheme [87]. Then, the CPMD runs were performed. They were divided into equilibation and production runs. Electronic exchange and correlation effects were treated using the PBE [88] and OLYP [89,90] functionals in both phases. The simulations were performed using pseudopotentials proposed by N. Troullier and J. L. Martins [91]. The dispersion corrections by Grimme (DFT-D2 method) were included to reproduce weak interactions in the studied structures [92]. The kinetic energy cutoff of 100 Ry was used to determine the upper limit of the plane-wave basis set. The fictitious orbital mass parameter was equal to 400 a.u. The time step was set to 3 a.u. Molecular dynamics simulations were carried out at 300 K temperature controlled by the Nosé–Hoover chains thermostat [93,94]. The initial equilibration period of the CPMD runs was accrued for 30,000 steps for the gas and crystalline phases and was excluded from the further trajectories analyses. The trajectories were collected for 24 ps in both phases.

In the next step of the investigatiob of molecular dynamics, nuclear quantum effects were modeled within the Path Integrals Molecular Dynamics (PIMD) approach [5,6] for the studied cationic forms of the proton sponges in the gas phase. The electronic structure was propagated according to the Car–Parrinello scheme with the same computational setup as given above for the CPMD runs with the PBE functional. Eight Trotter replicas and staging transformation were used as parts of the PIMD setup. A separate Nosé–Hoover chains thermostat was coupled with each degree of freedom (“massive” thermostatting), and the simulations were carried out at 100 K and 300 K. As in the CPMD run, the first 30,000 steps were taken as equilibration phase—a time much larger than needed for the convergence of the primitive and virial kinetic energy estimators—and the PIMD data collection lasted for a further 12 ps.

In addition, metadynamics [78] based on the CPMD computational setup was employed to study the proton potential profile in the N-H⋯N hydrogen-bonded moiety. The gas phase models of the studied “proton sponges” were investigated via metadynamics at 100 K and 300 K lasting for 24 ps. The N–H, H⋯N, and N⋯N interatomic distances were taken as collective variables (CVs). The time-dependent metadynamics potential consisted of Gaussian hills with an initial height of 0.001 a.u. and transversal width of 0.05 a.u., placed every 150–500 CPMD steps (depending on the actual displacement of the CVs from the previous position). The 3D volumetric free energy maps were then prepared using the Vreco utility written by Nisanth Nair and distributed along with the CPMD package [85].

The CPMD study was followed by analysis of snapshots from the dynamics to characterize the intramolecular hydrogen bonding. The Quantum Theory of Atoms in Molecules (QTAIM) [95] and Density Overlap Regions Indicator (DORI) [82] approaches were used to obtain an in-depth characterization of the intramolecular hydrogen bridge in the studied compounds. For this purpose, four snapshots of the CPMD trajectory in the gas phase for each compound and each temperature were taken (at 0, 8, 16, and 24 ps of simulation time) and analyzed, yielding 32 snapshots in total. This allowed us to track changes in the electron structure during the simulation time. Kohn–Sham equations were solved for the snapshot structures at the PBE0-D3/def2-TZVP level [88,96,97], and the corresponding wavefunctions were saved for subsequent QTAIM processing. These electronic structure calculations were carried out with the Gaussian16 Rev. C.01 program [98]. The QTAIM analysis (determination of electron density critical points and their local properties) was carried out with assistance of the AimAll [99] software, while the DORI surfaces were generated with the MultiWFN program [83,84]. The QTAIM studies were carried out at non-equilibrium structures, but the usual nomenclature, associated with standard equilibrium-structure QTAIM calculations, was retained for convenience, following the practice common in the literature [100,101,102,103]. In addition, the proton reaction path within the intramolecular hydrogen bridges was studied by means of the scan with geometry optimization approach (with 0.05 Å increment of the N-H distance, freezing of the NHN valence angle describing the HB and optimization of the remaining structural parameters of the investigated compounds) using the PBE0-D3/def2-TZVP level of theory. The data were analyzed and visualized using the VMD [104], Vesta [105], Chimera [106], Gnuplot [107], and GaussView6 [108] programs.

## 4. Conclusions

In the present work, we focused on characterizing intra- and intermolecular interactions (N-H⋯N) in symmetric and asymmetric “proton sponges” based on theoretical methods. The Hirshfeld surface and fingerprint analyses were applied to give insight into intra- and intermolecular interactions in the crystal structures of the studied compounds. The CPMD results obtained for the gas phase as well as crystalline phase revealed the time-evolution in hydrogen bridge metric parameters. Metadynamics simulations were employed to reconstruct free energy surfaces in the investigated “proton sponges”. In addition, the QTAIM and DORI approaches made it possible to determine the strength and nature of interactions based on electron density. Therefore, based on the research methods used and the results obtained, we came to the following conclusions:

1. The Hirshfeld Surface (HS) and fingerprint data show intra- and intermolecular interactions in the crystals of the studied “proton sponges”.
2. Car–Parrinello Molecular Dynamics results enabled the time-evolution analysis of the bridged proton in the intramolecular hydrogen bond. The proton is very labile, and its movement is governed by a double-well potential, which can be asymmetric. The broken symmetry in the symmetric “proton sponges” can have internal origin (not enough energy to cross the barriers-vanishing at 300 K) or external cause (asymmetry of the crystal environment). For the asymmetric compounds (3) and (4), the intramolecular factor is more important than the environment. Quantum effects reproduced via the PIMD approach lead to significant broadening of the bridge proton probability distribution.
3. Metadynamics has allowed us to estimate the depths of free energy wells, ranging from −17.7 to −34 kcal/mol. This biased molecular dynamics scheme has proven successful in investigating the double-well free energy surface, but the proton remained within the space between the two nitrogen atoms.
4. QTAIM studies showed that the stronger H⋯N interactions are covalent in the investigated compounds (1)–(3), while the weaker contacts exhibit only subtle covalency. A different situation is observed for compound (4) with the supremacy of the H⋯N interactions with the proton directed to the “donor” nitrogen atom of the amine group of the substituted ring.
5. DORI molecular plots revealed regions indicating the presence of covalent and non-covalent interactions for compound (4) at 100 K with simulation times of 0 and 24 ps.

## Figures and Tables

**Table 1:** The interatomic contacts present in the crystal structures of the investigated “proton sponges”. Symbol “+” means the presence of the contact, while “−” means the absence of the contact.

Compound	Contact				
	*O⋯H*	*Cl⋯H*	*C⋯H*	*H⋯Br*	*C-H⋯π*
**(1)**	**+**	**+**	−	−	−
**(2)**	**+**	−	**+**	−	**+**
**(3)**	**+**	−	**+**	**+**	**+**
**(4)**	**+**	−	−	−	−

**Table 2:** Proton possession percentage during the CPMD simulation of the DMANH${}^{+}$ and its derivatives. The reference nitrogen atoms are those at the position 1 of the aromatic system, i.e., the left amine functional group in Figure 1. The simulations were carried out using the PBE functional.

Compound	Proton Possession (%)			
	Gas Phase		Crystalline Phase	
	100 K	300 K	100 K	300 K
**(1)**	50.2	48.9	84.4	60.8
**(2)**	51.8	50.8	29.7	48.4
**(3)**	91.5	74.2	55.4	72.5
**(4)**	18.3	37.8	10.9	36.8

**Table 3:** Cambridge Crystallographic Data Centre (CCDC) code, number, and experimental unit cell data for the investigated “proton sponges”.

Number	CCDC Code and Number	Unit Cell Data	Ref.
1	**TAPCES** (1266338)	orthorhombic, a = 11.363 Å, b = 16.676 Å,	[47]
		c = 20.307 Å, Z = 4	
2	**RISBEA** (122006)	tetragonal, a = 11.410 Å, b = 11.410 Å,	[48]
		c = 13.126 Å, Z = 4	
3	**XUCKAH** (170012)	monoclinic, a = 11.182 Å, b = 14.236 Å,	[49]
		c = 19.935 Å, Z = 8	
4	**ZOSKEX** (1315231)	triclinic, a= 7.986 Å, b = 12.463 Å,	[50]
		c = 8.663 Å, Z = 2	

## Data Availability

The data gathered in this study are presented in the manuscript and the Supplementary Material.

## Supplementary Materials

The following supporting information can be downloaded at: https://www.mdpi.com/article/10.3390/ijms24021542/s1.

## Abbreviations

The following abbreviations are used in this manuscript:

**BCP**	Bond Critical Point
CPMD	Car–Parrinello Molecular Dynamics
CVs	Collective Variables
DFT	Density Functional Theory
DORI	Density Overlap Regions Indicator
HB	Hydrogen Bond
IR	Infrared Spectroscopy
LBHB	Low-Barrier Hydrogen Bond
MD	Molecular Dynamics
NCI	Non-covalent Interactions Index
NMR	Nuclear magnetic resonance
PIMD	Path Integrals Molecular Dynamics
PT	Proton transfer
QTAIM	Quantum Theory of Atoms in Molecules
RCP	Ring Critical Point
RDG	Reduced Density Gradient
VDW	van der Waals
