# Supplementary material for: Unraveling the Nature of Hydrogen Bonds of “Proton Sponges” Based on Car-Parrinello and Metadynamics Approaches
Source: Int J Mol Sci. 2023 Jan 12;24(2):1542. doi: 10.3390/ijms24021542 (PMC9860969; doi:10.3390/ijms24021542)
Supplement: Supplementary file 1 [file ijms-24-01542-s001.zip › ijms-2062984-supplementary.pdf]

Supplementary Information

Unraveling Nature of Hydrogen Bonds of "Proton Sponges" based on Car-Parrinello and metadynamics approaches

Beata Kizior [a], Mariusz, Michalczyk [a], Jarosław J. Panek [b], Wiktor Zierkiewicz [a] and Aneta Jezierska [b]*

[a] Faculty of Chemistry, Wrocław University of Science and Technology, ul. Wybrzeże Wyspiańskiego 27, 50-370 Wroclaw, Poland
[b] Faculty of Chemistry, University of Wrocław, ul. F. Joliot-Curie 14, 50-383 Wrocław, Poland, Tel: +(+48) (71) (3757 224); Fax: +(48) (71) (3282 348)
e-mail: aneta.jezierska@chem.uni.wroc.pl (A. Jezierska)

*Corresponding author: Aneta Jezierska, aneta.jezierska@chem.uni.wroc.pl

phone. +48 71 3757 224; fax: +48 71 3282 348

Table of contents:

1. **Figure S1.** Molecular structures of the studied protonated form of 1,8-bis(dimethylamino)naphthalene ($DMANH^+$) and its derivatives obtained as a result of CPMD geometry optimization with PBE functional. Color coding: red - oxygen; blue - nitrogen; gray - carbon; white - hydrogen. The solid line between nitrogen atoms indicates the presence of intramolecular hydrogen bonding (HB).
2. **Figure S2.** Crystal unit cells of the studied compounds (suitable references are included in the manuscript). The dotted lines indicate the presence of intramolecular hydrogen bridges. The data were used to construct CPMD models for crystalline phase simulations. Color coding: red - oxygen; blue - nitrogen; gray - carbon; white – hydrogen, green – chlorine, orange – bromine.
3. **Figure S3.** Complete 2-D fingerprint plot based on Hirshfeld surface of the studied compounds (1)-(4) calculated from their experimental crystal structures. The X and Y axes correspond respectively to d_i and d_e, where d_i (d_e) is the distance from a point on the surface to the nearest nucleus inside (outside) the surface. The color scale shows the values of the d_{norm} Hirshfeld normalized contact distance.

4. **Figure S4.** Time-evolution of metric parameters of atoms involved in the intramolecular hydrogen bond N-H···N. The CPMD results in the gas phase at 100 K for $DMANH^+$ and its derivatives. The calculations were carried out using the OLYP functional.
5. **Figure S5.** Time-evolution of metric parameters of atoms involved in the intramolecular hydrogen bond N-H···N. The CPMD results in the gas phase at 300 K for $DMANH^+$ and its derivatives. The calculations were carried out using the OLYP functional.
6. **Figure S6.** Time-evolution of metric parameters of atoms involved in the intramolecular hydrogen bond N-H···N. The CPMD results in the crystalline phase at 100 K for $DMANH^+$ and its derivatives. The calculations were carried out using the OLYP functional.
7. **Figure S7.** Time-evolution of metric parameters of atoms involved in the intramolecular hydrogen bond N-H···N. The CPMD results in the crystalline phase at 300 K for $DMANH^+$ and its derivatives. The calculations were carried out using the OLYP functional.
8. **Table S1.** The proton possession percentage during the CPMD simulation of the $DMANH^+$ and its derivatives. The reference nitrogen atoms are those at the position 1 of the aromatic system, i.e. the left-side amine functions in Figure 1 in the manuscript. The calculations were carried out using the OLYP functional. The main manuscript body contains corresponding data for the PBE functional.
9. **Figure S8.** Potential energy profile obtained as a result of a scan with optimization method for the investigated "proton sponges". The simulations were performed at the PBE0-D3/def2-TZVP level of theory.
10. **Table S2.** QTAIM parameters for BCPs of N···H and H···N interactions (presented one above the other in single table cell) within studied compounds in the gas phase. All QTAIM data are given in a.u.
11. XYZ coordinates.

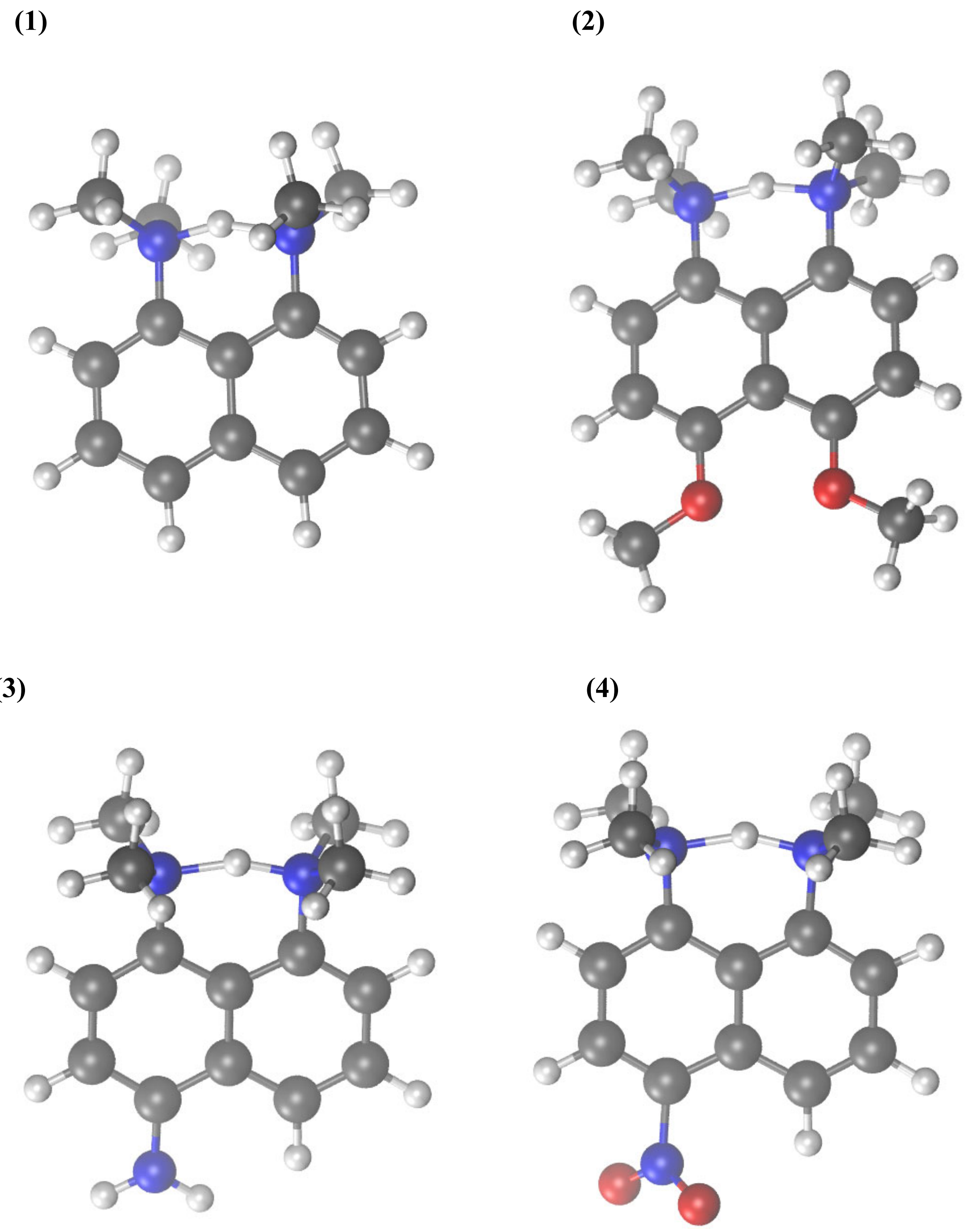

Figure S1. Molecular structures of the studied protonated form of 1,8-bis(dimethylamino)naphthalene (DMANH$^+$) and its derivatives obtained as a result of CPMD geometry optimization with PBE functional. Color coding: red - oxygen; blue - nitrogen; gray - carbon; white - hydrogen. The solid line between nitrogen atoms indicates the presence of intramolecular hydrogen bonding (HB).

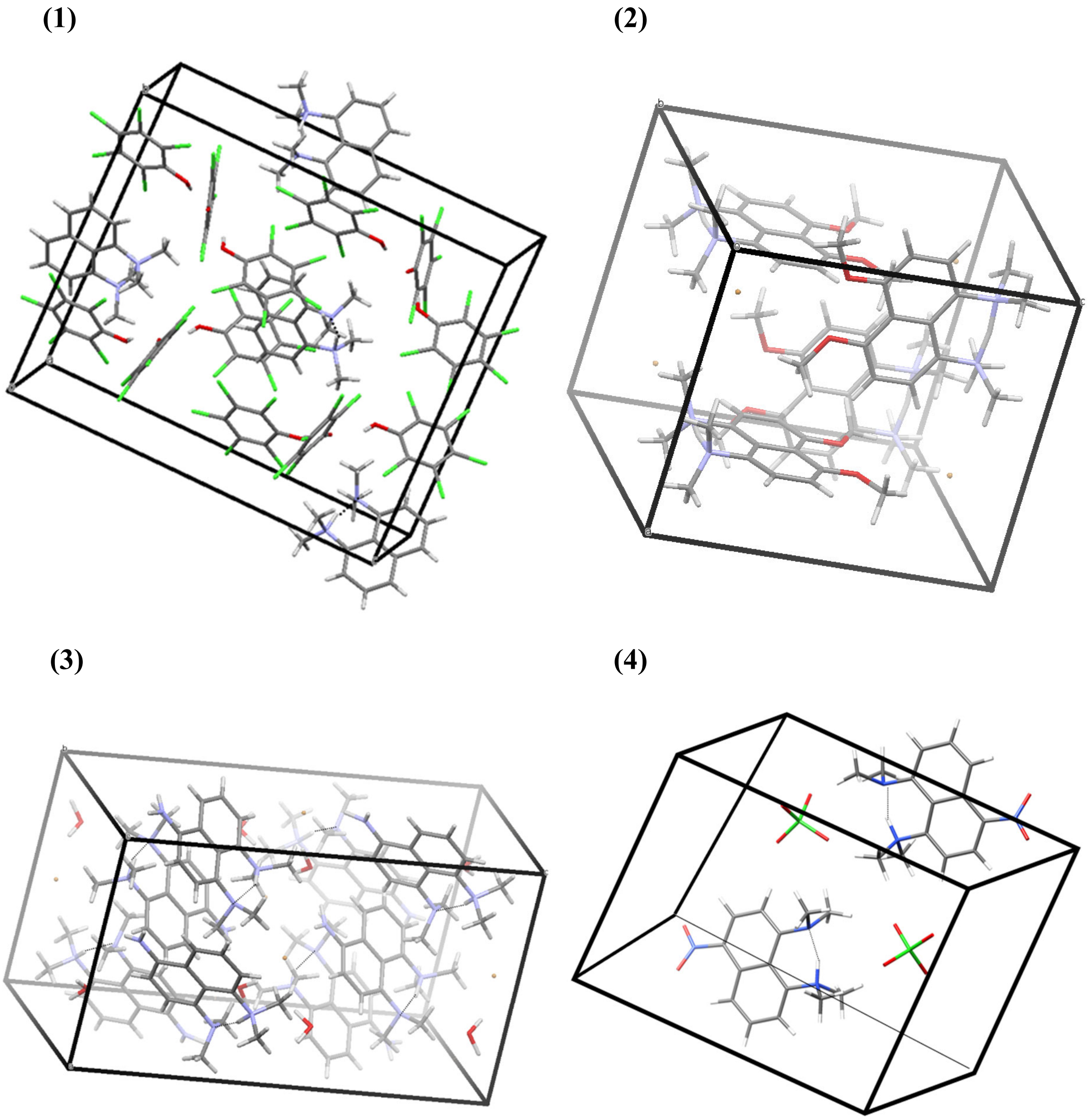

Figure S2. Crystal unit cells of the studied compounds (suitable references are included in the manuscript). The dotted lines indicate the presence of intramolecular hydrogen bridges. The data were used to construct CPMD models for crystalline phase simulations. Color coding: red - oxygen; blue - nitrogen; gray - carbon; white – hydrogen, green – chlorine, orange – bromine.

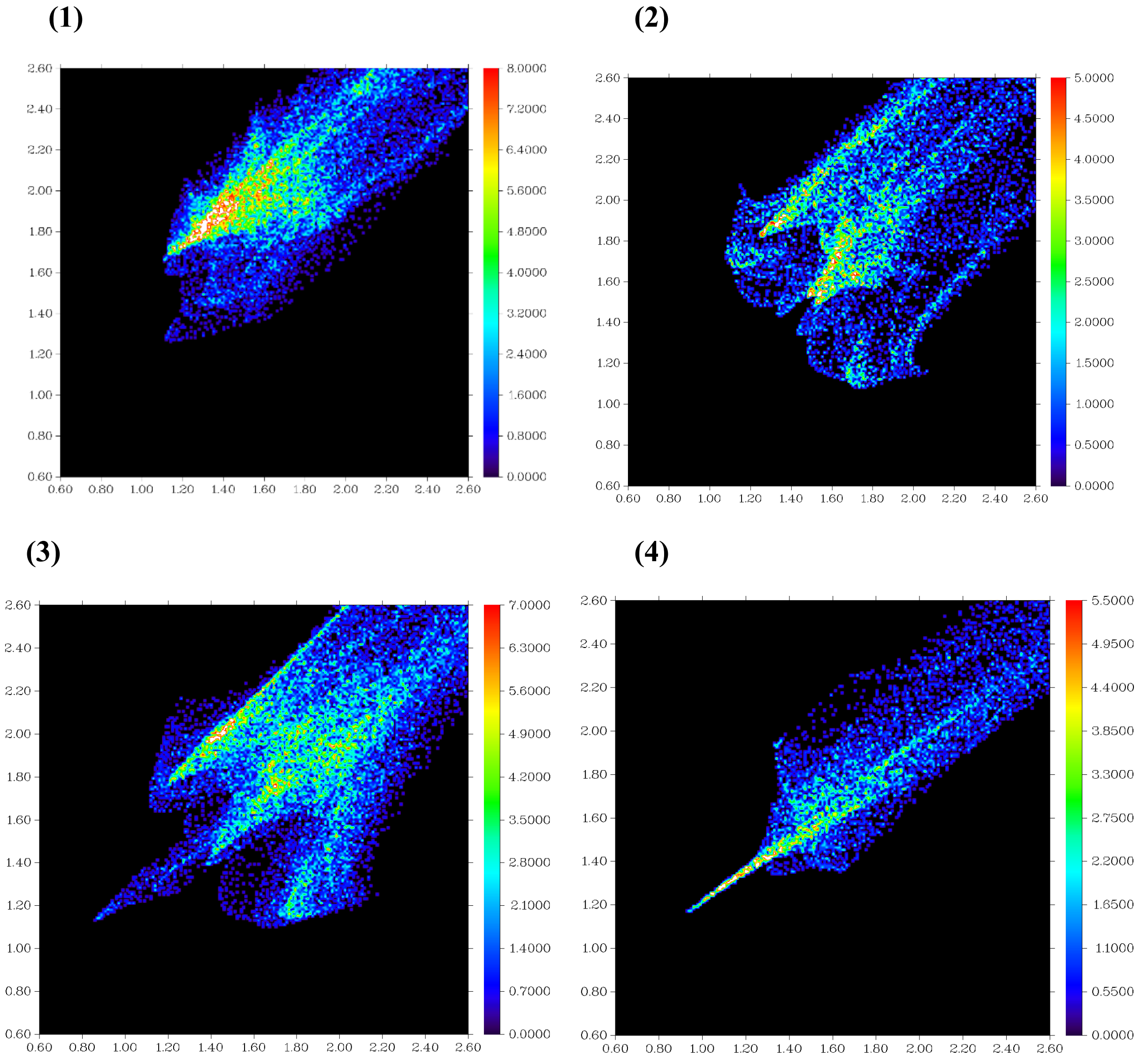

Figure S3. Complete 2-D fingerprint plot based on Hirshfeld surface of the studied compounds (1)-(4) calculated from their experimental crystal structures. The X and Y axes correspond respectively to d_i and d_e, where d_i (d_e) is the distance from a point on the surface to the nearest nucleus inside (outside) the surface. The color scale shows the values of the d_{norm} Hirshfeld normalized contact distance.

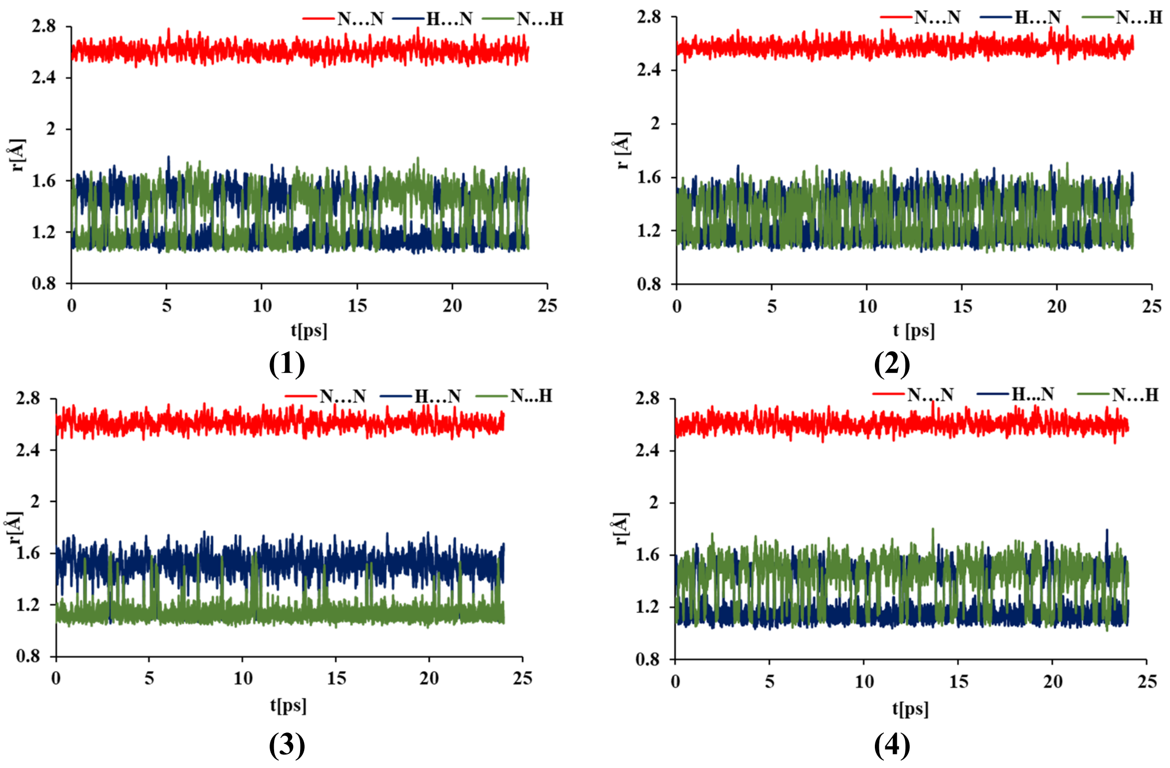

Figure S4. Time-evolution of metric parameters of atoms involved in the intramolecular hydrogen bond N-H···N. The CPMD results in the gas phase at 100 K for $DMANH^+$ and its derivatives. The calculations were carried out using the OLYP functional.

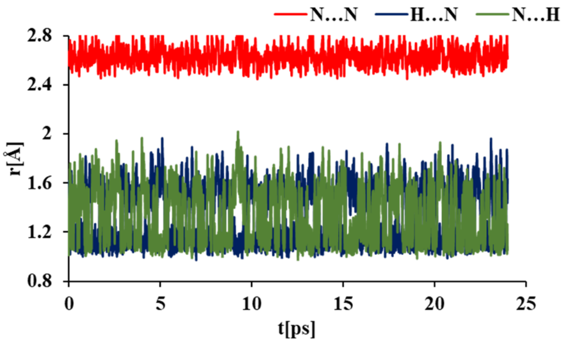

(1)

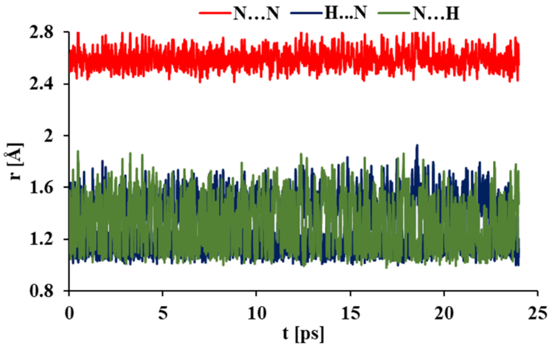

(2)

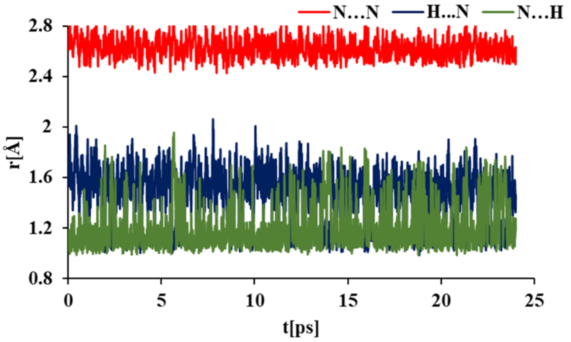

(3)

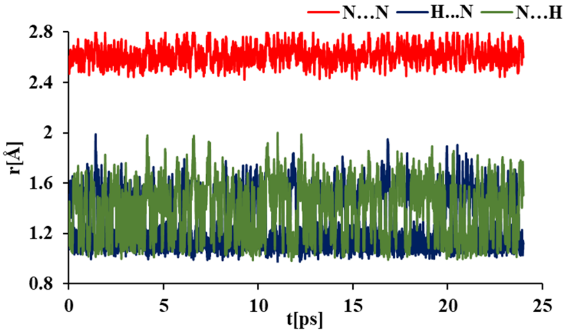

(4)

Figure S5. Time-evolution of metric parameters of atoms involved in the intramolecular hydrogen bond N-H···N. The CPMD results in the gas phase at 300 K for $DMANH^+$ and its derivatives. The calculations were carried out using the OLYP functional.

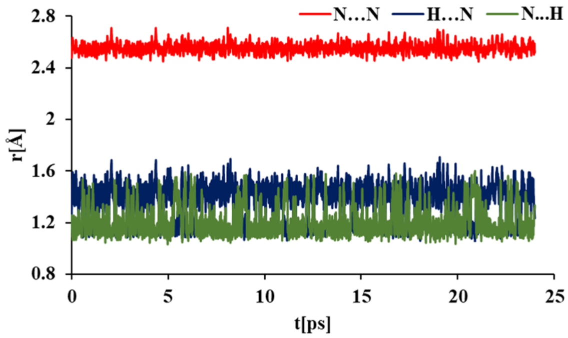

(1)

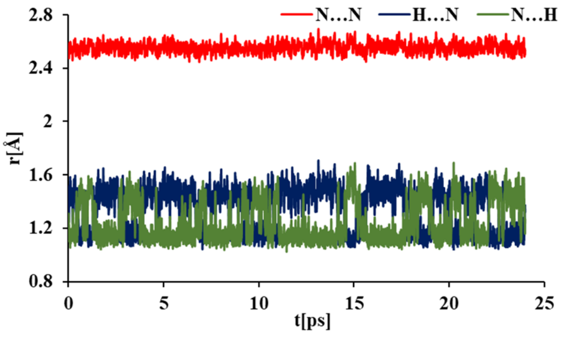

(2)

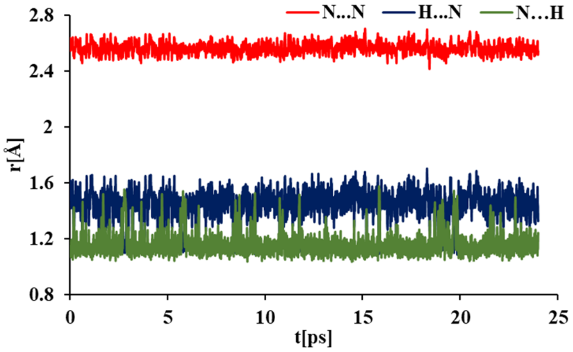

(3)

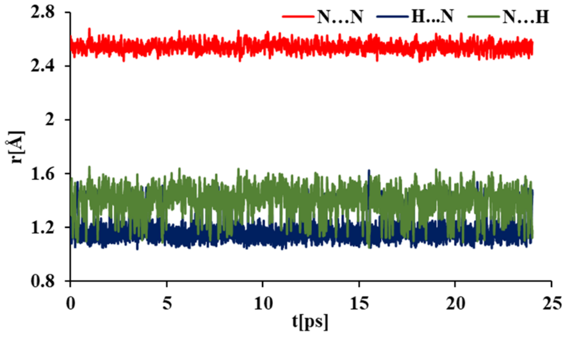

(4)

Figure S6. Time-evolution of metric parameters of atoms involved in the intramolecular hydrogen bond N-H···N. The CPMD results in the crystalline phase at 100 K for $DMANH^+$ and its derivatives. The calculations were carried out using the OLYP functional.

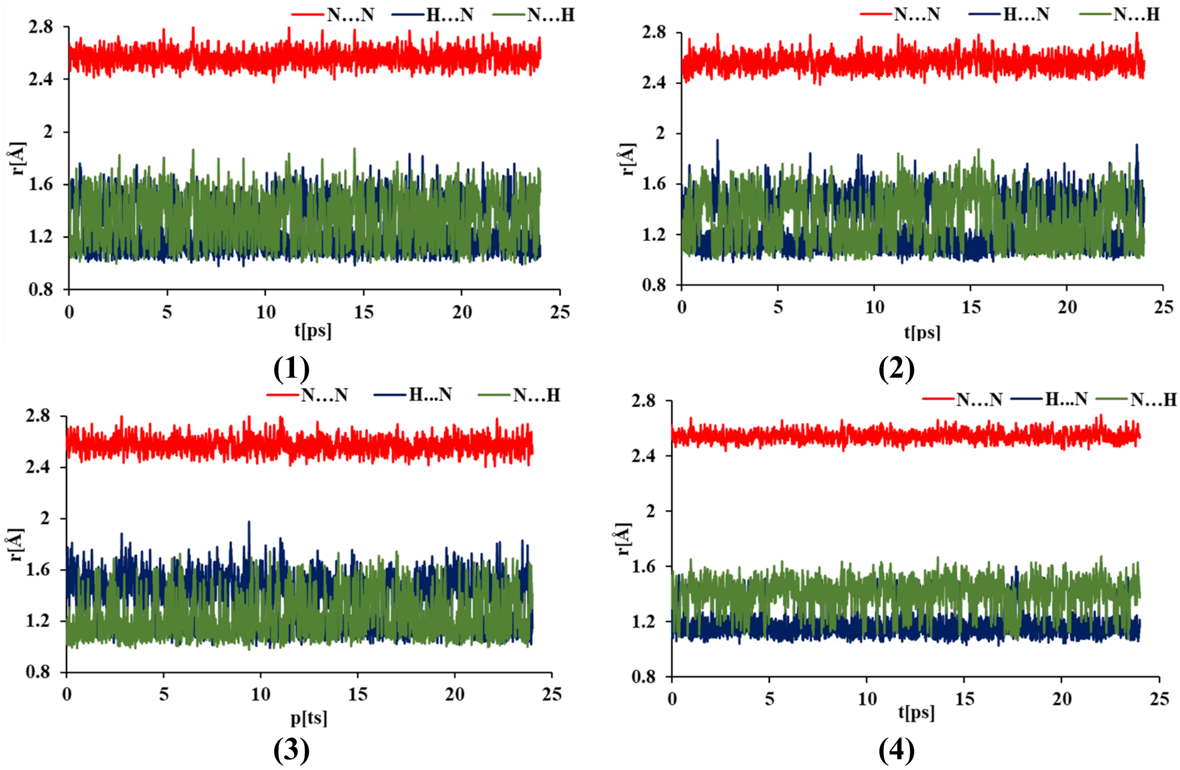

Figure S7. Time-evolution of metric parameters of atoms involved in the intramolecular hydrogen bond N-H···N. The CPMD results in the crystalline phase at 300 K for $DMANH^+$ and its derivatives. The calculations were carried out using the OLYP functional.

Table S1. The proton possession percentage during the CPMD simulation of the $DMANH^+$ and its derivatives. The reference nitrogen atoms are those at the position 1 of the aromatic system, i.e. the left-side amine functions in Figure 1 in the manuscript. The calculations were carried out using the OLYP functional. The main manuscript body contains corresponding data for the PBE functional.

Compound	Proton possession (%)			
	Gas phase		Crystalline phase	
	100 K	300 K	100 K	300 K
**(1)**	44.0	48.4	76.9	59.5
**(2)**	48.6	47.9	33.4	54.7
**(3)**	93.7	75.0	91.6	66.0
**(4)**	26.4	38.2	14.6	15.1

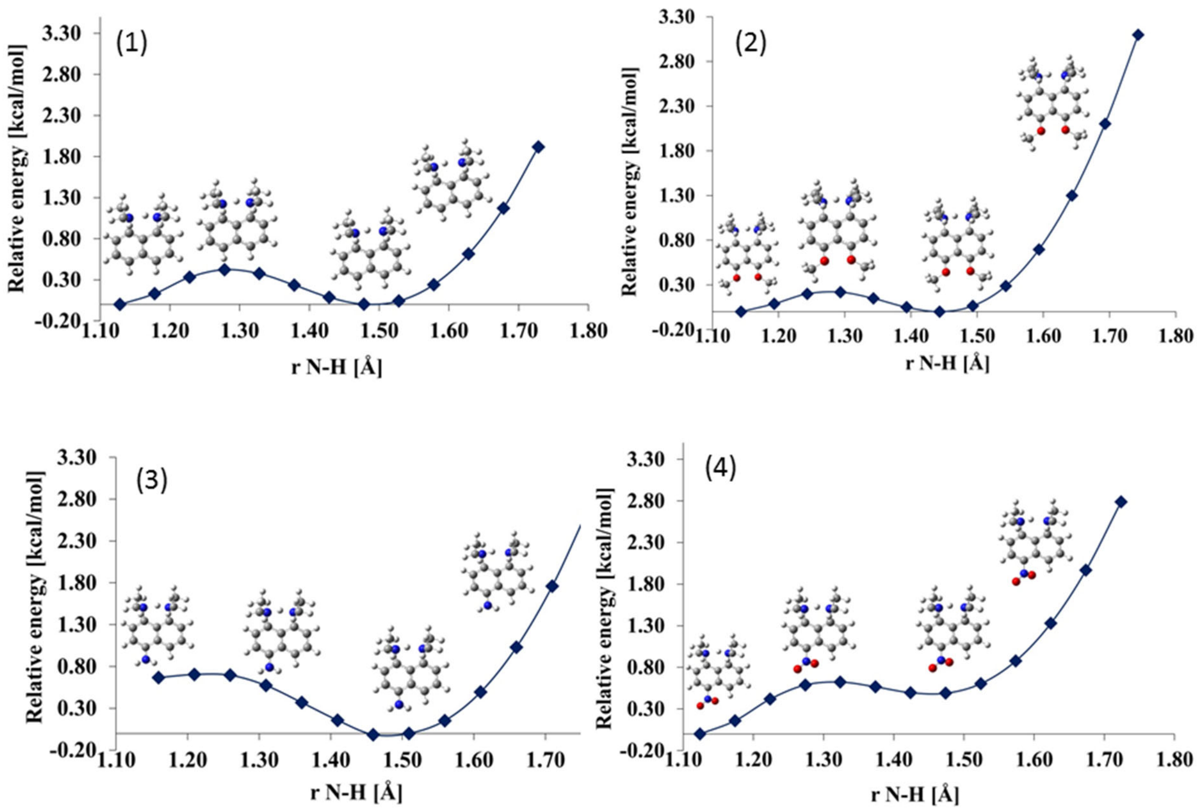

Figure S8. Potential energy profile obtained as a result of a scan with optimization method for the investigated "proton sponges". The simulations were performed at the PBE0-D3/def2-TZVP level of theory.

Table S2. QTAIM parameters for BCPs of N···H and H···N interactions (presented one above the other in single table cell) within studied compounds in the gas phase. All QTAIM data are given in a.u.

Compound	Snapshot time [ps]	100 K						300 K					
		ρ	$\nabla^2\rho$	ε	H	V	G	ρ	$\nabla^2\rho$	ε	H	V	G
**(1)**	**0**	0.228<br>0.103	-0.779<br>0.023	0.005<br>0.019	-0.253<br>-0.049	-0.312<br>-0.105	0.059<br>0.055	0.121<br>0.202	-0.026<br>-0.511	0.016<br>0.004	-0.067<br>-0.187	-0.127<br>-0.247	0.060<br>0.060
	**8**	0.229<br>0.090	-0.798<br>0.033	0.006<br>0.019	-0.253<br>-0.039	-0.307<br>-0.087	0.053<br>0.048	0.308<br>0.080	-1.670<br>0.099	0.004<br>0.025	-0.478<br>-0.029	-0.539<br>-0.082	0.061<br>0.054
	**16**	0.140<br>0.199	-0.079<br>-0.479	0.013<br>0.005	-0.087<br>-0.187	-0.155<br>-0.254	0.067<br>0.067	0.080<br>0.266	0.062<br>-1.173	0.021<br>0.006	-0.031<br>-0.349	-0.077<br>-0.405	0.046<br>0.056
	**24**	0.208<br>0.132	-0.564<br>-0.053	0.009<br>0.010	-0.208<br>-0.078	-0.276<br>-0.143	0.067<br>0.065	0.091<br>0.237	0.041<br>-0.867	0.017<br>0.007	-0.039<br>-0.272	-0.089<br>-0.328	0.050<br>0.056
**(2)**	**0**	0.104<br>0.225	0.018<br>-0.752	0.019<br>0.008	-0.050<br>-0.247	-0.104<br>-0.306	0.054<br>0.059	0.104<br>0.225	0.018<br>-0.752	0.019<br>0.008	-0.050<br>-0.247	-0.104<br>-0.305	0.054<br>0.058
	**8**	0.231<br>0.111	-0.795<br>0.014	0.005<br>0.021	-0.261<br>-0.056	-0.324<br>-0.116	0.063<br>0.060	0.111<br>0.210	-0.003<br>-0.610	0.027<br>0.006	-0.057<br>-0.210	-0.113<br>-0.268	0.056<br>0.058
	**16**	0.211<br>0.125	-0.591<br>-0.028	0.006<br>0.015	-0.213<br>-0.070	-0.278<br>-0.133	0.065<br>0.063	0.210<br>0.133	-0.581<br>-0.048	0.004<br>0.015	-0.214<br>-0.079	-0.283<br>-0.146	0.069<br>0.067
	**24**	0.139<br>0.171	-0.122<br>-0.298	0.014<br>0.012	-0.088<br>-0.135	-0.146<br>-0.196	0.058<br>0.061	0.206<br>0.150	-0.516<br>-0.103	0.011<br>0.016	-0.202<br>-0.101	-0.276<br>-0.175	0.073<br>0.075
**(3)**	**0**	0.163<br>0.171	-0.211<br>-0.267	0.008<br>0.011	-0.122<br>-0.135	-0.191<br>-0.203	0.069<br>0.068	0.116<br>0.226	0.003<br>-0.745	0.015<br>0.010	-0.061<br>-0.250	-0.123<br>-0.314	0.062<br>0.064
	**8**	0.159<br>0.159	-0.203<br>-0.210	0.010<br>0.011	-0.114<br>-0.114	-0.178<br>-0.177	0.063<br>0.062	0.163<br>0.129	-0.273<br>-0.105	0.013<br>0.020	-0.124<br>-0.077	-0.180<br>-0.129	0.056<br>0.051
	**16**	0.090<br>0.241	0.044<br>-0.922	0.019<br>0.005	-0.039<br>-0.286	-0.089<br>-0.342	0.050<br>0.056	0.153<br>0.168	-0.169<br>-0.259	0.010<br>0.015	-0.106<br>-0.129	-0.170<br>-0.193	0.064<br>0.064
	**24**	0.107<br>0.217	0.011<br>-0.675	0.022<br>0.004	-0.053<br>-0.227	-0.109<br>-0.285	0.056<br>0.058	0.058<br>0.287	0.078<br>-1.400	0.030<br>0.003	-0.016<br>-0.400	-0.051<br>-0.450	0.035<br>0.050
**(4)**	**0**	0.103<br>0.229	0.022<br>-0.781	0.019<br>0.006	-0.050<br>-0.254	-0.105<br>-0.313	0.055<br>0.059	0.216<br>0.130	-0.612<br>-0.033	0.004<br>0.018	-0.222<br>-0.075	-0.291<br>-0.141	0.069<br>0.066
	**8**	0.097<br>0.238	0.034<br>-0.886	0.025<br>0.006	-0.044<br>-0.280	-0.097<br>-0.338	0.052<br>0.058	0.108<br>0.242	0.030<br>-0.911	0.017<br>0.008	-0.054<br>-0.291	-0.114<br>-0.355	0.060<br>0.064
	**16**	0.112<br>0.213	-0.007<br>-0.635	0.011<br>0.009	-0.058<br>-0.219	-0.115<br>-0.279	0.057<br>0.060	0.252<br>0.087	-1.024<br>0.055	0.001<br>0.028	-0.312<br>-0.036	-0.368<br>-0.085	0.056<br>0.050
	**24**	0.227<br>0.092	-0.788<br>0.031	0.004<br>0.016	-0.251<br>-0.041	-0.305<br>-0.090	0.054<br>0.049	0.064<br>0.305	0.092<br>-1.627	0.020<br>0.004	-0.019<br>-0.461	-0.060<br>-0.515	0.042<br>0.054

XYZ coordinates

The models for CPMD and DFT simulations were prepared based on X-ray data. The xyz experimental coordinates are provided below for the studied compounds. Suitable references for the crystal structures are cited in the manuscript.

Compound (1) CCDC identifiers: TAPCES (1266338)

XYZ for single molecule:

35			
TAPCES			
N	0.384524	0.462592	6.779898
H	-0.386342	-0.336855	6.849551
N	-1.303904	-1.354425	7.393169
C	0.497699	0.827797	8.204231
C	1.210160	1.920241	8.599202
C	1.355606	2.201732	9.968706
C	0.836317	1.356426	10.900391
C	-0.414749	-0.680214	11.492341
C	-1.111301	-1.791336	11.128642
C	-1.380604	-2.035973	9.768073
C	-0.922676	-1.182162	8.802881
C	-0.121584	-0.056365	9.133479
C	0.103403	0.208283	10.522884
C	-0.052270	1.566210	5.903651
C	1.627182	-0.175432	6.276284
C	-0.993126	-2.696509	6.859908
C	-2.725984	-0.991388	7.191115
H	1.636272	2.474718	7.988774
H	1.818080	2.931641	10.253004
H	0.943129	1.582552	11.824766
H	-0.227260	-0.453587	12.425853
H	-1.443101	-2.396341	11.806490
H	-1.874895	-2.841590	9.491492
H	-0.874951	1.941086	6.213942
H	-0.181808	1.200672	5.015829
H	0.636328	2.227914	5.864662
H	2.340778	0.473598	6.220034
H	1.931710	-0.928853	6.884073
H	1.443101	-0.565316	5.387447
H	-1.590820	-3.381893	7.261783

H	-1.272656	-2.689839	5.933705
H	0.022726	-2.841590	7.030283
H	-2.897565	-0.001668	7.479068
H	-3.408900	-1.614237	7.631371
H	-2.931654	-1.023906	6.244402

XYZ for crystal:

292

TAPCES

N	11.747524	17.138592	6.779898
H	10.976658	16.339145	6.849551
N	10.059096	15.321575	7.393169
C	11.860699	17.503797	8.204231
C	12.573160	18.596241	8.599202
C	12.718606	18.877732	9.968706
C	12.199317	18.032426	10.900391
C	10.948251	15.995786	11.492341
C	10.251699	14.884664	11.128642
C	9.982396	14.640027	9.768073
C	10.440324	15.493838	8.802881
C	11.241416	16.619635	9.133479
C	11.466403	16.884283	10.522884
C	11.310730	18.242210	5.903651
C	12.990182	16.500568	6.276284
C	10.369874	13.979491	6.859908
C	8.637016	15.684612	7.191115
H	12.999272	19.150718	7.988774
H	13.181080	19.607641	10.253004
H	12.306129	18.258552	11.824766
H	11.135740	16.222413	12.425853
H	9.919899	14.279659	11.806490
H	9.488105	13.834410	9.491492
H	10.488049	18.617086	6.213942
H	11.181192	17.876672	5.015829
H	11.999328	18.903914	5.864662
H	13.703778	17.149598	6.220034
H	13.294710	15.747147	6.884073
H	12.806101	16.110684	5.387447
H	9.772180	13.294107	7.261783
H	10.090344	13.986161	5.933705

H	11.385726	13.834410	7.030283
H	8.465435	16.674332	7.479068
H	7.954100	15.061763	7.631371
H	8.431346	15.652094	6.244402
N	6.066024	7.875408	13.527102
H	5.295158	8.674855	13.457449
N	4.377596	9.692425	12.913831
C	6.179199	7.510203	12.102769
C	6.891660	6.417759	11.707798
C	7.037106	6.136268	10.338294
C	6.517817	6.981574	9.406609
C	5.266751	9.018214	8.814659
C	4.570199	10.129336	9.178358
C	4.300896	10.373973	10.538927
C	4.758824	9.520162	11.504119
C	5.559916	8.394365	11.173521
C	5.784903	8.129717	9.784116
C	5.629230	6.771790	14.403349
C	7.308682	8.513432	14.030716
C	4.688374	11.034509	13.447092
C	2.955516	9.329388	13.115885
H	7.317772	5.863282	12.318226
H	7.499580	5.406359	10.053996
H	6.624629	6.755448	8.482234
H	5.454240	8.791587	7.881147
H	4.238399	10.734341	8.500510
H	3.806605	11.179590	10.815508
H	4.806549	6.396914	14.093058
H	5.499692	7.137328	15.291171
H	6.317828	6.110086	14.442338
H	8.022278	7.864402	14.086966
H	7.613210	9.266853	13.422927
H	7.124601	8.903316	14.919553
H	4.090680	11.719893	13.045217
H	4.408844	11.027839	14.373295
H	5.704226	11.179590	13.276717
H	2.783935	8.339668	12.827932
H	2.272600	9.952237	12.675629
H	2.749846	9.361906	14.062597
N	-0.384524	8.800592	3.373602

H	0.386342	8.001145	3.303949
N	1.303904	6.983575	2.760331
C	-0.497699	9.165797	1.949269
C	-1.210159	10.258241	1.554298
C	-1.355606	10.539732	0.184794
C	-0.836317	9.694426	-0.746891
C	0.414750	7.657786	-1.338841
C	1.111301	6.546664	-0.975142
C	1.380605	6.302027	0.385427
C	0.922676	7.155838	1.350619
C	0.121584	8.281635	1.020021
C	-0.103403	8.546283	-0.369384
C	0.052270	9.904210	4.249849
C	-1.627182	8.162568	3.877216
C	0.993126	5.641491	3.293592
C	2.725984	7.346612	2.962385
H	-1.636272	10.812718	2.164726
H	-1.818080	11.269641	-0.099504
H	-0.943129	9.920552	-1.671266
H	0.227260	7.884413	-2.272353
H	1.443101	5.941659	-1.652990
H	1.874895	5.496410	0.662008
H	0.874951	10.279086	3.939558
H	0.181808	9.538672	5.137671
H	-0.636328	10.565914	4.288838
H	-2.340778	8.811598	3.933466
H	-1.931710	7.409147	3.269427
H	-1.443101	7.772684	4.766053
H	1.590820	4.956107	2.891717
H	1.272656	5.648161	4.219795
H	-0.022726	5.496410	3.123217
H	2.897565	8.336332	2.674432
H	3.408900	6.723763	2.522129
H	2.931654	7.314094	3.909097
N	5.296976	-0.462592	16.933398
H	6.067842	0.336855	17.003051
N	6.985404	1.354425	17.546669
C	5.183801	-0.827797	18.357731
C	4.471341	-1.920241	18.752702
C	4.325894	-2.201732	20.122206

C	4.845183	-1.356426	21.053891
C	6.096250	0.680214	21.645841
C	6.792801	1.791336	21.282142
C	7.062105	2.035973	19.921573
C	6.604176	1.182162	18.956381
C	5.803084	0.056365	19.286979
C	5.578097	-0.208283	20.676384
C	5.733770	-1.566210	16.057151
C	4.054318	0.175432	16.429784
C	6.674626	2.696509	17.013408
C	8.407484	0.991388	17.344615
H	4.045228	-2.474718	18.142274
H	3.863420	-2.931641	20.406504
H	4.738371	-1.582552	21.978266
H	5.908760	0.453587	22.579353
H	7.124601	2.396341	21.959990
H	7.556395	2.841590	19.644992
H	6.556451	-1.941086	16.367442
H	5.863308	-1.200672	15.169329
H	5.045172	-2.227914	16.018162
H	3.340722	-0.473598	16.373534
H	3.749790	0.928853	17.037573
H	4.238399	0.565316	15.540947
H	7.272320	3.381893	17.415283
H	6.954156	2.689839	16.087205
H	5.658774	2.841590	17.183783
H	8.579065	0.001668	17.632568
H	9.090400	1.614237	17.784871
H	8.613154	1.023906	16.397902
O	9.100172	3.940205	4.941708
C	7.816608	3.867331	5.105586
C	7.032561	4.951771	5.561478
C	5.655365	4.899075	5.684132
C	4.973926	3.721249	5.393742
C	5.705362	2.604458	5.005472
C	7.079149	2.686504	4.845047
Cl	7.878763	6.409421	5.969649
Cl	4.761665	6.297858	6.165408
Cl	3.248795	3.652044	5.514772
Cl	4.894612	1.097614	4.770520

Cl	7.986144	1.308566	4.317471
O	3.418672	4.397795	15.365292
C	2.135108	4.470669	15.201414
C	1.351061	3.386229	14.745522
C	-0.026135	3.438925	14.622868
C	-0.707574	4.616751	14.913258
C	0.023862	5.733542	15.301528
C	1.397649	5.651496	15.461953
Cl	2.197263	1.928579	14.337351
Cl	-0.919835	2.040142	14.141592
Cl	-2.432705	4.685956	14.792228
Cl	-0.786888	7.240386	15.536480
Cl	2.304644	7.029434	15.989529
O	2.262828	12.278205	5.211792
C	3.546392	12.205331	5.047914
C	4.330439	13.289771	4.592022
C	5.707635	13.237075	4.469368
C	6.389074	12.059249	4.759758
C	5.657638	10.942458	5.148028
C	4.283851	11.024504	5.308453
Cl	3.484237	14.747421	4.183851
Cl	6.601335	14.635858	3.988092
Cl	8.114205	11.990044	4.638728
Cl	6.468388	9.435614	5.382980
Cl	3.376856	9.646566	5.836029
O	7.944328	12.735795	15.095208
C	9.227892	12.808669	15.259086
C	10.011939	11.724229	15.714978
C	11.389135	11.776925	15.837632
C	12.070574	12.954751	15.547242
C	11.339138	14.071542	15.158972
C	9.965351	13.989496	14.998547
Cl	9.165737	10.266579	16.123149
Cl	12.282835	10.378142	16.318908
Cl	13.795705	13.023956	15.668272
Cl	12.149888	15.578386	14.924020
Cl	9.058356	15.367434	14.470971
O	10.845529	2.822413	3.274504
H	10.181248	3.176778	3.529357
C	11.016429	2.765214	1.948863

C	10.302832	3.529475	1.023473
C	10.580089	3.437257	-0.343594
C	11.534581	2.546258	-0.803954
C	12.252723	1.782831	0.107424
C	12.019781	1.914572	1.466572
Cl	9.094150	4.601075	1.616640
Cl	9.729228	4.458495	-1.451747
Cl	11.841155	2.399343	-2.498979
Cl	13.423907	0.642360	-0.446145
Cl	12.970524	1.040249	2.607825
O	5.164029	5.515587	17.032496
H	4.499748	5.161222	16.777643
C	5.334929	5.572786	18.358137
C	4.621332	4.808525	19.283527
C	4.898589	4.900743	20.650594
C	5.853081	5.791742	21.110954
C	6.571223	6.555169	20.199576
C	6.338281	6.423428	18.840428
Cl	3.412650	3.736925	18.690360
Cl	4.047728	3.879505	21.758747
Cl	6.159655	5.938657	22.805979
Cl	7.742407	7.695640	20.753145
Cl	7.289024	7.297751	17.699175
O	0.517471	11.160413	6.878996
H	1.181752	11.514778	6.624143
C	0.346572	11.103214	8.204637
C	1.060168	11.867475	9.130027
C	0.782911	11.775257	10.497094
C	-0.171581	10.884258	10.957454
C	-0.889723	10.120831	10.046076
C	-0.656781	10.252572	8.686928
Cl	2.268850	12.939075	8.536860
Cl	1.633772	12.796495	11.605247
Cl	-0.478155	10.737343	12.652479
Cl	-2.060907	8.980360	10.599645
Cl	-1.607524	9.378249	7.545675
O	6.198971	13.853587	13.428004
H	6.863252	13.499222	13.682857
C	6.028072	13.910786	12.102363
C	6.741668	13.146525	11.176973

C	6.464411	13.238743	9.809906
C	5.509919	14.129742	9.349546
C	4.791777	14.893169	10.260924
C	5.024719	14.761428	11.620072
Cl	7.950350	12.074925	11.770140
Cl	7.315272	12.217505	8.701753
Cl	5.203345	14.276657	7.654521
Cl	3.620593	16.033640	9.707355
Cl	4.073976	15.635751	12.761325
C	11.010747	5.356498	7.638478
O	10.893367	5.101689	6.339033
H	10.203974	4.754328	6.083977
C	11.932286	6.346719	8.021671
C	12.170909	6.649722	9.353404
C	11.492538	5.968007	10.357585
C	10.574408	4.983956	10.009930
C	10.317604	4.697129	8.671089
Cl	12.755990	7.197028	6.766902
Cl	13.319709	7.871072	9.756295
Cl	11.753660	6.363728	12.016464
Cl	9.780248	4.080617	11.243986
Cl	9.134375	3.518136	8.242408
C	5.329247	2.981502	12.668522
O	5.211867	3.236311	13.967967
H	4.522474	3.583672	14.223023
C	6.250786	1.991281	12.285329
C	6.489409	1.688278	10.953596
C	5.811038	2.369993	9.949415
C	4.892908	3.354044	10.297070
C	4.636104	3.640871	11.635911
Cl	7.074490	1.140972	13.540098
Cl	7.638209	0.466928	10.550705
Cl	6.072160	1.974272	8.290536
Cl	4.098748	4.257383	9.063014
Cl	3.452875	4.819864	12.064592
C	0.352253	13.694498	2.515022
O	0.469633	13.439689	3.814467
H	1.159026	13.092328	4.069523
C	-0.569286	14.684719	2.131829
C	-0.807909	14.987722	0.800096

C	-0.129538	14.306007	-0.204085
C	0.788592	13.321956	0.143570
C	1.045396	13.035129	1.482411
Cl	-1.392990	15.535028	3.386598
Cl	-1.956709	16.209072	0.397205
Cl	-0.390660	14.701728	-1.862964
Cl	1.582752	12.418617	-1.090486
Cl	2.228625	11.856136	1.911092
C	6.033753	11.319502	17.791978
O	6.151133	11.574311	16.492533
H	6.840526	11.921672	16.237477
C	5.112214	10.329281	18.175171
C	4.873591	10.026278	19.506904
C	5.551962	10.707993	20.511085
C	6.470092	11.692044	20.163430
C	6.726896	11.978871	18.824589
Cl	4.288510	9.478972	16.920402
Cl	3.724791	8.804928	19.909795
Cl	5.290840	10.312272	22.169964
Cl	7.264252	12.595383	21.397486
Cl	7.910125	13.157864	18.395908

Compound (2) CCDC identifiers: RISBEA (122006)

XYZ for single molecule:

43

odorf4

C	4.108741	2.852500	9.664674
C	5.266856	2.852500	8.987372
C	5.281689	2.852500	7.578952
C	4.104177	2.852500	6.888525
C	2.852500	2.852500	7.560576
C	2.852500	2.852500	9.009686
N	4.128138	2.852500	11.136098
O	4.050550	2.852500	5.531296
C	4.744278	1.631630	11.690016
C	5.280548	2.852500	4.810679
H	2.852500	2.852500	11.248982
H	6.138580	2.852500	9.490098
H	6.195630	2.852500	7.061788
H	4.643870	1.540350	12.600960

H	4.153240	0.855750	11.445872
H	5.796280	1.506120	11.590258
H	5.921790	1.939700	5.119140
H	5.100270	2.852500	3.977178
C	1.596259	2.852500	9.664674
C	0.438144	2.852500	8.987372
C	0.423311	2.852500	7.578952
C	1.600823	2.852500	6.888525
N	1.576862	2.852500	11.136098
O	1.654450	2.852500	5.531296
C	0.960722	4.073370	11.690016
C	0.424452	2.852500	4.810679
H	-0.433580	2.852500	9.490098
H	-0.490630	2.852500	7.061788
H	1.061130	4.164650	12.600960
H	1.551760	4.849250	11.445872
H	-0.091280	4.198880	11.590258
H	-0.216790	3.765300	5.119140
H	0.604730	2.852500	3.977178
C	4.744278	4.073370	11.690016
H	4.643870	4.164650	12.600960
H	4.153240	4.849250	11.445872
H	5.796280	4.198880	11.590258
H	5.921790	3.765300	5.119140
C	0.960722	1.631630	11.690016
H	1.061130	1.540350	12.600960
H	1.551760	0.855750	11.445872
H	-0.091280	1.506120	11.590258
H	-0.216790	1.939700	5.119140

XYZ for crystal:

132

odorf4

Br	2.852500	2.852500	1.702442
Br	8.557500	2.852500	11.423558
Br	2.852500	8.557500	11.423558
C	4.108741	2.852500	9.664674
C	5.266856	2.852500	8.987372
C	5.281689	2.852500	7.578952
C	4.104177	2.852500	6.888525

C	2.852500	2.852500	7.560576
C	2.852500	2.852500	9.009686
N	4.128138	2.852500	11.136098
O	4.050550	2.852500	5.531296
C	4.744278	1.631630	11.690016
C	5.280548	2.852500	4.810679
H	2.852500	2.852500	11.248982
H	6.138580	2.852500	9.490098
H	6.195630	2.852500	7.061788
H	4.643870	1.540350	12.600960
H	4.153240	0.855750	11.445872
H	5.796280	1.506120	11.590258
H	5.921790	1.939700	5.119140
H	5.100270	2.852500	3.977178
C	1.596259	2.852500	9.664674
C	0.438144	2.852500	8.987372
C	0.423311	2.852500	7.578952
C	1.600823	2.852500	6.888525
N	1.576862	2.852500	11.136098
O	1.654450	2.852500	5.531296
C	0.960722	4.073370	11.690016
C	0.424452	2.852500	4.810679
H	-0.433580	2.852500	9.490098
H	-0.490630	2.852500	7.061788
H	1.061130	4.164650	12.600960
H	1.551760	4.849250	11.445872
H	-0.091280	4.198880	11.590258
H	-0.216790	3.765300	5.119140
H	0.604730	2.852500	3.977178
C	4.744278	4.073370	11.690016
H	4.643870	4.164650	12.600960
H	4.153240	4.849250	11.445872
H	5.796280	4.198880	11.590258
H	5.921790	3.765300	5.119140
C	0.960722	1.631630	11.690016
H	1.061130	1.540350	12.600960
H	1.551760	0.855750	11.445872
H	-0.091280	1.506120	11.590258
H	-0.216790	1.939700	5.119140
C	8.557500	4.108741	3.461326

C	8.557500	5.266856	4.138628
C	8.557500	5.281689	5.547048
C	8.557500	4.104177	6.237475
C	8.557500	2.852500	5.565424
C	8.557500	2.852500	4.116314
N	8.557500	4.128138	1.989902
O	8.557500	4.050550	7.594704
C	9.778370	4.744278	1.435984
C	8.557500	5.280548	8.315321
H	8.557500	2.852500	1.877018
H	8.557500	6.138580	3.635902
H	8.557500	6.195630	6.064212
H	9.869650	4.643870	0.525040
H	10.554250	4.153240	1.680128
H	9.903880	5.796280	1.535742
H	9.470300	5.921790	8.006860
H	8.557500	5.100270	9.148822
C	8.557500	1.596259	3.461326
C	8.557500	0.438144	4.138628
C	8.557500	0.423311	5.547048
C	8.557500	1.600823	6.237475
N	8.557500	1.576862	1.989902
O	8.557500	1.654450	7.594704
C	7.336630	0.960722	1.435984
C	8.557500	0.424452	8.315321
H	8.557500	-0.433580	3.635902
H	8.557500	-0.490630	6.064212
H	7.245350	1.061130	0.525040
H	6.560750	1.551760	1.680128
H	7.211120	-0.091280	1.535742
H	7.644700	-0.216790	8.006860
H	8.557500	0.604730	9.148822
C	7.336630	4.744278	1.435984
H	7.245350	4.643870	0.525040
H	6.560750	4.153240	1.680128
H	7.211120	5.796280	1.535742
H	7.644700	5.921790	8.006860
C	9.778370	0.960722	1.435984
H	9.869650	1.061130	0.525040
H	10.554250	1.551760	1.680128

H	9.903880	-0.091280	1.535742
H	9.470300	-0.216790	8.006860
C	2.852500	7.301259	3.461326
C	2.852500	6.143144	4.138628
C	2.852500	6.128311	5.547048
C	2.852500	7.305823	6.237475
C	2.852500	8.557500	5.565424
C	2.852500	8.557500	4.116314
N	2.852500	7.281862	1.989902
O	2.852500	7.359450	7.594704
C	1.631630	6.665722	1.435984
C	2.852500	6.129452	8.315321
H	2.852500	8.557500	1.877018
H	2.852500	5.271420	3.635902
H	2.852500	5.214370	6.064212
H	1.540350	6.766130	0.525040
H	0.855750	7.256760	1.680128
H	1.506120	5.613720	1.535742
H	1.939700	5.488210	8.006860
H	2.852500	6.309730	9.148822
C	2.852500	9.813741	3.461326
C	2.852500	10.971856	4.138628
C	2.852500	10.986689	5.547048
C	2.852500	9.809177	6.237475
N	2.852500	9.833138	1.989902
O	2.852500	9.755550	7.594704
C	4.073370	10.449278	1.435984
C	2.852500	10.985548	8.315321
H	2.852500	11.843580	3.635902
H	2.852500	11.900630	6.064212
H	4.164650	10.348870	0.525040
H	4.849250	9.858240	1.680128
H	4.198880	11.501280	1.535742
H	3.765300	11.626790	8.006860
H	2.852500	10.805270	9.148822
C	4.073370	6.665722	1.435984
H	4.164650	6.766130	0.525040
H	4.849250	7.256760	1.680128
H	4.198880	5.613720	1.535742
H	3.765300	5.488210	8.006860

C	1.631630	10.449278	1.435984
H	1.540350	10.348870	0.525040
H	0.855750	9.858240	1.680128
H	1.506120	11.501280	1.535742
H	1.939700	11.626790	8.006860

Compound (3) CCDC identifiers: XUCKAH (170012)

XYZ for single molecule:

37

brom

N	6.868125	-0.872809	7.632716
N	8.264594	0.976447	6.535100
N	4.820869	0.833660	2.052970
C	5.976222	-0.893167	6.484306
C	4.912783	-1.741490	6.438672
H	4.726012	-2.294843	7.162677
C	4.104064	-1.779785	5.304151
H	3.391371	-2.375988	5.273794
C	4.333426	-0.971038	4.246019
H	3.778545	-1.022145	3.501974
C	5.656047	0.848750	3.145427
C	6.693551	1.712448	3.216260
H	6.846176	2.299114	2.509913
C	7.536921	1.743341	4.316456
H	8.253205	2.337551	4.321416
C	7.346926	0.931319	5.383714
C	6.264553	0.005694	5.398198
C	5.408362	-0.043420	4.253956
C	6.190314	-0.439892	8.891244
H	6.835930	-0.384372	9.599178
H	5.512297	-1.077665	9.124973
H	5.787323	0.421386	8.757911
C	7.576674	-2.163730	7.790652
H	8.180658	-2.108352	8.535689
H	8.071697	-2.357482	6.990059
H	6.937363	-2.862860	7.948389
C	8.256635	2.295270	7.249780
H	8.837713	2.249288	8.011881
H	7.364048	2.496994	7.539660
H	8.563919	2.983866	6.654742

C	9.639365	0.538121	6.174982
H	10.172490	0.468364	6.970218
H	10.035044	1.178741	5.579349
H	9.598763	-0.317463	5.742047
H	7.561197	-0.024201	7.329344
H	4.872382	1.383739	1.523805
H	4.208140	0.000000	1.914677

XYZ for crystal:

342

brom

Br	4.110120	3.559427	19.659089
Br	4.174684	10.677427	10.102728
Br	5.140416	10.676573	0.182122
Br	5.075852	3.558573	9.738483
Br	0.000000	0.000000	0.000000
Br	-1.931464	0.000000	19.841211
Br	0.000000	14.236000	0.000000
Br	-1.931464	14.236000	19.841211
Br	11.182000	0.000000	0.000000
Br	9.250536	0.000000	19.841211
Br	11.182000	14.236000	0.000000
Br	9.250536	14.236000	19.841211
Br	-0.965732	7.118000	9.920606
Br	10.216268	7.118000	9.920606
Br	0.000000	7.118000	0.000000
Br	-1.931464	7.118000	19.841211
Br	11.182000	7.118000	0.000000
Br	9.250536	7.118000	19.841211
Br	-0.965732	0.000000	9.920606
Br	-0.965732	14.236000	9.920606
Br	10.216268	0.000000	9.920606
Br	10.216268	14.236000	9.920606
O	2.807740	12.935114	1.310115
H	3.370620	12.351154	0.765871
H	1.995325	13.135557	0.684522
O	7.408528	5.817114	8.610491
H	6.845648	5.233154	9.154735
H	8.220943	6.017557	9.236084
O	6.442796	1.300886	18.531096

H	5.879917	1.884846	19.075341
H	7.255211	1.100443	19.156690
O	1.842009	8.418886	11.230721
H	2.404888	9.002846	10.686476
H	1.029593	8.218443	10.605128
O	8.593044	5.193578	1.381742
H	9.297527	5.613255	0.853172
H	8.190540	4.595381	0.680554
O	1.623224	12.311578	8.538864
H	0.918741	12.731255	9.067434
H	2.025729	11.713381	9.240052
O	0.657492	9.042422	18.459470
H	-0.046991	8.622745	18.988039
H	1.059997	9.640619	19.160658
O	7.627312	1.924422	11.302348
H	8.331795	1.504745	10.773778
H	7.224808	2.522619	10.601159
N	6.868125	-0.872809	7.632716
N	8.264594	0.976447	6.535100
N	4.820869	0.833660	2.052970
C	5.976222	-0.893167	6.484306
C	4.912783	-1.741490	6.438672
H	4.726012	-2.294843	7.162677
C	4.104064	-1.779785	5.304151
H	3.391371	-2.375988	5.273794
C	4.333426	-0.971038	4.246019
H	3.778545	-1.022145	3.501974
C	5.656047	0.848750	3.145427
C	6.693551	1.712448	3.216260
H	6.846176	2.299114	2.509913
C	7.536921	1.743341	4.316456
H	8.253205	2.337551	4.321416
C	7.346926	0.931319	5.383714
C	6.264553	0.005694	5.398198
C	5.408362	-0.043420	4.253956
C	6.190314	-0.439892	8.891244
H	6.835930	-0.384372	9.599178
H	5.512297	-1.077665	9.124973
H	5.787323	0.421386	8.757911
C	7.576674	-2.163730	7.790652

H	8.180658	-2.108352	8.535689
H	8.071697	-2.357482	6.990059
H	6.937363	-2.862860	7.948389
C	8.256635	2.295270	7.249780
H	8.837713	2.249288	8.011881
H	7.364048	2.496994	7.539660
H	8.563919	2.983866	6.654742
C	9.639365	0.538121	6.174982
H	10.172490	0.468364	6.970218
H	10.035044	1.178741	5.579349
H	9.598763	-0.317463	5.742047
H	7.561197	-0.024201	7.329344
H	4.872382	1.383739	1.523805
H	4.208140	0.000000	1.914677
N	3.348143	6.245191	2.287890
N	1.951674	8.094447	3.385506
N	5.395399	7.951660	7.867636
C	4.240046	6.224833	3.436299
C	5.303485	5.376510	3.481934
H	5.490257	4.823157	2.757928
C	6.112204	5.338215	4.616455
H	6.824897	4.742012	4.646812
C	5.882842	6.146962	5.674586
H	6.437723	6.095855	6.418632
C	4.560221	7.966750	6.775178
C	3.522717	8.830448	6.704345
H	3.370092	9.417114	7.410692
C	2.679347	8.861341	5.604150
H	1.963064	9.455551	5.599190
C	2.869342	8.049319	4.536891
C	3.951716	7.123694	4.522407
C	4.807907	7.074580	5.666650
C	4.025954	6.678108	1.029362
H	3.380338	6.733628	0.321428
H	4.703972	6.040335	0.795633
H	4.428945	7.539386	1.162695
C	2.639595	4.954270	2.129954
H	2.035610	5.009648	1.384917
H	2.144571	4.760518	2.930547
H	3.278905	4.255140	1.972216

C	1.959633	9.413270	2.670825
H	1.378555	9.367288	1.908725
H	2.852220	9.614994	2.380945
H	1.652349	10.101866	3.265863
C	0.576903	7.656121	3.745624
H	0.043779	7.586364	2.950388
H	0.181224	8.296741	4.341257
H	0.617505	6.800537	4.178559
H	2.655071	7.093799	2.591262
H	5.343887	8.501739	8.396801
H	6.008128	7.118000	8.005929
N	2.382411	15.108809	12.208496
N	0.985942	13.259553	13.306112
N	4.429667	13.402340	17.788241
C	3.274314	15.129167	13.356905
C	4.337753	15.977490	13.402540
H	4.524525	16.530843	12.678534
C	5.146472	16.015785	14.537060
H	5.859165	16.611988	14.567417
C	4.917110	15.207038	15.595192
H	5.471991	15.258145	16.339238
C	3.594489	13.387250	16.695784
C	2.556985	12.523552	16.624951
H	2.404360	11.936886	17.331298
C	1.713615	12.492659	15.524756
H	0.997332	11.898449	15.519796
C	1.903610	13.304681	14.457497
C	2.985984	14.230306	14.443013
C	3.842175	14.279420	15.587256
C	3.060222	14.675892	10.949968
H	2.414607	14.620372	10.242033
H	3.738240	15.313665	10.716238
H	3.463213	13.814614	11.083301
C	1.673863	16.399730	12.050560
H	1.069878	16.344352	11.305522
H	1.178839	16.593482	12.851153
H	2.313173	17.098860	11.892822
C	0.993901	11.940730	12.591431
H	0.412823	11.986712	11.829330
H	1.886488	11.739006	12.301551

**H**	**0.686618**	**11.252134**	**13.186469**
**C**	**-0.388829**	**13.697879**	**13.666230**
**H**	**-0.921953**	**13.767636**	**12.870994**
**H**	**-0.784508**	**13.057259**	**14.261863**
**H**	**-0.348227**	**14.553463**	**14.099165**
**H**	**1.689339**	**14.260201**	**12.511868**
**H**	**4.378155**	**12.852261**	**18.317406**
**H**	**5.042396**	**14.236000**	**17.926535**
**N**	**5.902393**	**7.990809**	**17.553321**
**N**	**7.298862**	**6.141553**	**16.455706**
**N**	**3.855138**	**6.284340**	**11.973576**
**C**	**5.010490**	**8.011167**	**16.404912**
**C**	**3.947052**	**8.859490**	**16.359277**
**H**	**3.760280**	**9.412843**	**17.083283**
**C**	**3.138332**	**8.897785**	**15.224757**
**H**	**2.425639**	**9.493988**	**15.194400**
**C**	**3.367694**	**8.089038**	**14.166625**
**H**	**2.812814**	**8.140145**	**13.422580**
**C**	**4.690316**	**6.269250**	**13.066033**
**C**	**5.727819**	**5.405552**	**13.136866**
**H**	**5.880444**	**4.818886**	**12.430519**
**C**	**6.571189**	**5.374659**	**14.237061**
**H**	**7.287473**	**4.780449**	**14.242022**
**C**	**6.381195**	**6.186681**	**15.304320**
**C**	**5.298821**	**7.112306**	**15.318804**
**C**	**4.442630**	**7.161420**	**14.174561**
**C**	**5.224583**	**7.557892**	**18.811849**
**H**	**5.870198**	**7.502372**	**19.519784**
**H**	**4.546565**	**8.195665**	**19.045579**
**H**	**4.821592**	**6.696614**	**18.678516**
**C**	**6.610942**	**9.281730**	**17.711257**
**H**	**7.214926**	**9.226352**	**18.456295**
**H**	**7.105965**	**9.475482**	**16.910665**
**H**	**5.971631**	**9.980860**	**17.868995**
**C**	**7.290904**	**4.822730**	**17.170386**
**H**	**7.871981**	**4.868712**	**17.932487**
**H**	**6.398316**	**4.621006**	**17.460266**
**H**	**7.598187**	**4.134134**	**16.575348**
**C**	**8.673634**	**6.579879**	**16.095588**
**H**	**9.206758**	**6.649636**	**16.890823**

H	9.069312	5.939259	15.499954
H	8.633031	7.435463	15.662652
H	6.595465	7.142201	17.249949
H	3.906650	5.734261	11.444411
H	3.242408	7.118000	11.835283
N	1.395501	4.416007	7.694025
N	2.732783	2.590525	6.502560
N	-0.861841	2.820294	2.156541
C	0.471159	4.468538	6.543433
C	-0.563698	5.344906	6.548790
H	-0.694117	5.903669	7.279740
C	-1.434003	5.410961	5.461095
H	-2.152238	6.001898	5.476174
C	-1.233479	4.610756	4.377566
H	-1.801761	4.685068	3.644831
C	0.003920	2.796520	3.210506
C	1.041529	1.895950	3.234316
H	1.157120	1.313983	2.517850
C	1.920956	1.840430	4.308717
H	2.620760	1.228567	4.287686
C	1.787543	2.653733	5.392048
C	0.715911	3.594305	5.453555
C	-0.183329	3.672461	4.341455
C	0.758921	3.910629	8.928744
H	0.071044	4.519930	9.206322
H	1.419481	3.836602	9.621003
H	0.372806	3.047928	8.759895
C	2.109887	5.696962	7.916048
H	2.813603	5.564852	8.555530
H	1.493680	6.353527	8.249976
H	2.484651	6.001898	7.087281
C	2.762027	1.288927	7.167042
H	3.290292	1.349573	7.966246
H	3.148181	0.637773	6.577362
H	1.867851	1.024992	7.392835
C	4.089137	3.025150	6.089268
H	4.459293	2.383106	5.480143
H	4.653420	3.093483	6.863075
H	4.032841	3.880734	5.656729
H	1.904416	3.687124	7.545613

H	-0.882272	2.049984	1.367059
H	-1.369767	3.581778	2.009915
N	8.820767	11.534007	2.226581
N	7.483485	9.708525	3.418045
N	11.078110	9.938294	7.764064
C	9.745109	11.586538	3.377173
C	10.779966	12.462906	3.371815
H	10.910386	13.021669	2.640865
C	11.650272	12.528961	4.459511
H	12.368506	13.119898	4.444431
C	11.449747	11.728756	5.543039
H	12.018029	11.803068	6.275775
C	10.212348	9.914520	6.710099
C	9.174739	9.013950	6.686290
H	9.059148	8.431983	7.402756
C	8.295312	8.958430	5.611888
H	7.595508	8.346567	5.632920
C	8.428725	9.771733	4.528558
C	9.500357	10.712305	4.467050
C	10.399597	10.790461	5.579150
C	9.457347	11.028629	0.991862
H	10.145224	11.637930	0.714284
H	8.796787	10.954602	0.299602
H	9.843462	10.165928	1.160711
C	8.106381	12.814962	2.004558
H	7.402665	12.682852	1.365075
H	8.722588	13.471527	1.670630
H	7.731617	13.119898	2.833325
C	7.454241	8.406927	2.753563
H	6.925976	8.467573	1.954359
H	7.068087	7.755773	3.343244
H	8.348417	8.142992	2.527770
C	6.127131	10.143150	3.831338
H	5.756975	9.501106	4.440463
H	5.562848	10.211483	3.057531
H	6.183427	10.998734	4.263876
H	8.311852	10.805124	2.374993
H	11.098540	9.167984	8.553546
H	11.586035	10.699778	7.910691
N	7.855035	9.819993	12.147186

N	6.517753	11.645475	13.338651
N	10.112378	11.415706	17.684670
C	8.779377	9.767462	13.297778
C	9.814234	8.891094	13.292421
H	9.944654	8.332331	12.561471
C	10.684540	8.825039	14.380116
H	11.402774	8.234102	14.365037
C	10.484015	9.625244	15.463645
H	11.052297	9.550932	16.196381
C	9.246616	11.439480	16.630705
C	8.209008	12.340050	16.606896
H	8.093416	12.922017	17.323362
C	7.329580	12.395570	15.532494
H	6.629776	13.007433	15.553526
C	7.462993	11.582267	14.449164
C	8.534625	10.641695	14.387656
C	9.433865	10.563539	15.499756
C	8.491615	10.325371	10.912468
H	9.179492	9.716070	10.634889
H	7.831056	10.399398	10.220208
H	8.877730	11.188072	11.081317
C	7.140649	8.539038	11.925163
H	6.436933	8.671148	11.285681
H	7.756856	7.882473	11.591236
H	6.765885	8.234102	12.753931
C	6.488509	12.947073	12.674169
H	5.960244	12.886427	11.874965
H	6.102355	13.598227	13.263850
H	7.382685	13.211008	12.448376
C	5.161399	11.210850	13.751944
H	4.791244	11.852894	14.361069
H	4.597116	11.142517	12.978136
H	5.217695	10.355266	14.184482
H	7.346120	10.548876	12.295599
H	10.132808	12.186016	18.474152
H	10.620303	10.654222	17.831297
N	0.429769	2.701993	17.614631
N	1.767051	4.527475	16.423166
N	-1.827573	4.297706	12.077147
C	-0.494573	2.649462	16.464039

C	-1.529430	1.773094	16.469396
H	-1.659849	1.214331	17.200346
C	-2.399735	1.707039	15.381701
H	-3.117970	1.116102	15.396780
C	-2.199211	2.507244	14.298172
H	-2.767493	2.432932	13.565436
C	-0.961811	4.321480	13.131112
C	0.075797	5.222050	13.154922
H	0.191388	5.804017	12.438455
C	0.955224	5.277570	14.229323
H	1.655028	5.889433	14.208292
C	0.821811	4.464267	15.312653
C	-0.249821	3.523695	15.374161
C	-1.149061	3.445539	14.262061
C	-0.206811	3.207371	18.849349
H	-0.894688	2.598070	19.126928
H	0.453749	3.281398	19.541609
H	-0.592926	4.070072	18.680501
C	1.144155	1.421038	17.836654
H	1.847871	1.553148	18.476136
H	0.527948	0.764473	18.170581
H	1.518919	1.116102	17.007886
C	1.796296	5.829073	17.087648
H	2.324561	5.768427	17.886852
H	2.182449	6.480227	16.497967
H	0.902119	6.093008	17.313441
C	3.123405	4.092850	16.009874
H	3.493561	4.734894	15.400748
H	3.687689	4.024517	16.783681
H	3.067110	3.237266	15.577335
H	0.938684	3.430876	17.466218
H	-1.848004	5.068016	11.287665
H	-2.335499	3.536222	11.930520

Compound (4) CCDC identifiers: ZOSKEX (1315231)

XYZ for single molecule:

37

Zostex

C	6.39533	5.88652	6.54964
C	7.04515	4.75785	7.04101

**C**	**8.14411**	**4.96402**	**7.83895**
**C**	**8.64055**	**6.17052**	**8.18531**
**C**	**8.42638**	**8.66718**	**8.11925**
**C**	**7.75765**	**9.78446**	**7.67638**
**C**	**6.64319**	**9.65707**	**6.83140**
**C**	**6.20355**	**8.41140**	**6.45309**
**C**	**6.83928**	**7.20808**	**6.88885**
**C**	**8.00059**	**7.36918**	**7.74319**
**C**	**5.57855**	**5.03161**	**4.40835**
**C**	**4.10938**	**5.03553**	**6.36339**
**C**	**5.28640**	**8.87533**	**4.20026**
**C**	**3.77660**	**8.85427**	**6.16304**
**O**	**10.56401**	**7.08916**	**9.21450**
**O**	**10.08288**	**4.97835**	**9.50593**
**H**	**4.92788**	**7.18627**	**5.46792**
**H**	**6.68939**	**3.75766**	**6.78189**
**H**	**9.29713**	**8.76625**	**8.76050**
**H**	**8.09447**	**10.77531**	**7.97838**
**H**	**6.13042**	**10.55401**	**6.48314**
**H**	**6.42768**	**5.53536**	**3.93306**
**H**	**5.84514**	**3.97567**	**4.57248**
**H**	**4.71139**	**5.06933**	**3.73819**
**H**	**3.85959**	**5.56835**	**7.28815**
**H**	**3.23916**	**5.03666**	**5.69535**
**H**	**4.35228**	**3.99139**	**6.61581**
**H**	**6.19134**	**8.41777**	**3.78841**
**H**	**4.42757**	**8.65589**	**3.55590**
**H**	**5.42650**	**9.95994**	**4.26704**
**H**	**2.93526**	**8.62541**	**5.49890**
**H**	**3.62027**	**8.38724**	**7.14078**
**H**	**3.85864**	**9.93945**	**6.28713**
**H**	**8.44419**	**4.26160**	**8.41577**
**N**	**5.23746**	**5.71094**	**5.68036**
**N**	**5.02815**	**8.30776**	**5.55805**
**N**	**9.85837**	**6.09866**	**9.03654**

XYZ for crystal:

84

Zostex

O	9.797701	9.324569	8.587165
O	9.331256	7.347575	9.261835
N	4.424457	7.886197	5.356292
N	4.199215	10.443096	5.228901
N	9.073052	8.365513	8.632422
C	5.604818	8.058296	6.192715
C	6.297469	6.950912	6.614279
C	7.430160	7.114729	7.438969
C	7.838767	8.331544	7.797675
C	7.551586	10.835759	7.816113
C	6.869531	11.926084	7.384492
C	5.767505	11.790336	6.541365
C	5.350096	10.565199	6.138239
C	6.024429	9.367024	6.557288
C	7.174842	9.522321	7.410473
C	4.735157	7.290954	4.042991
C	3.350457	7.169006	6.067838
C	4.523592	10.973890	3.866152
C	2.959249	11.033991	5.782047
H	4.127759	9.475353	5.036976
H	5.917675	6.137639	6.419840
H	8.419168	10.814674	8.556993
H	7.197729	12.788968	7.685370
H	5.380697	12.543310	6.243838
H	5.484578	7.595377	3.687637
H	4.832538	6.408725	4.131829
H	4.052497	7.702081	3.494874
H	3.094817	7.484871	7.023272
H	2.646250	7.368345	5.456026
H	3.661597	6.352375	6.336030
H	5.293487	10.514185	3.536779
H	3.813612	10.634574	3.310492
H	4.593514	11.832717	3.905542
H	2.387514	10.801517	4.860976
H	2.838227	10.646095	6.713175
H	3.113486	11.888323	5.774504
O	-4.556257	2.816268	-0.206172

O	-4.089812	4.793262	-0.880843
N	0.816987	4.254641	3.024700
N	1.042229	1.697741	3.152091
N	-3.831608	3.775324	-0.251430
C	-0.363375	4.082541	2.188277
C	-1.056025	5.189925	1.766713
C	-2.188716	5.026108	0.942024
C	-2.597324	3.809293	0.583317
C	-2.310142	1.305079	0.564879
C	-1.628088	0.214753	0.996500
C	-0.526061	0.350501	1.839628
C	-0.108653	1.575639	2.242754
C	-0.782985	2.773814	1.823704
C	-1.933398	2.618517	0.970519
C	0.506286	4.849884	4.338002
C	1.890987	4.971831	2.313154
C	0.717852	1.166947	4.514841
C	2.282195	1.106847	2.598946
H	1.113685	2.665484	3.344016
H	-0.676231	6.003198	1.961151
H	-3.177725	1.326164	-0.176001
H	-1.956285	-0.648130	0.695622
H	-0.139254	-0.402473	2.137153
H	-0.243134	4.545460	4.693356
H	0.408906	5.732112	4.249163
H	1.188946	4.438756	4.886119
H	2.146627	4.655967	1.357721
H	2.595194	4.772492	2.924966
H	1.579847	5.788463	2.044962
H	-0.052044	1.626653	4.844214
H	1.427831	1.506263	5.070500
H	0.647930	0.308120	4.475450
H	2.853930	1.339320	3.520017
H	2.403217	1.494743	1.667817
H	2.127957	0.252515	2.606489
Cl	0.562549	9.158448	3.130302
O	-0.114074	10.089131	3.922304
O	-0.118291	8.335442	2.288012
O	1.451305	9.971679	2.380202
O	1.115541	8.327844	4.081544

Cl	4.678895	2.982389	5.250692
O	5.355518	2.051706	4.458688
O	5.359734	3.805396	6.092981
O	3.790138	2.169159	6.000791
O	4.125901	3.812994	4.299448
H	8.090280	6.380725	7.851740
H	-2.848836	5.760113	0.529254
